# Supplementary material for: 1000 Families Study, a UK multiwave cohort investigating the well-being of families of children with intellectual disabilities: cohort profile
Source: BMJ Open. 2020 Feb 12;10(2):e032919. doi: 10.1136/bmjopen-2019-032919 (PMC7045113; doi:10.1136/bmjopen-2019-032919)
Supplement: Author's manuscript [file bmjopen-2019-032919.draft_revisions.pdf]

BMJ Open is committed to open peer review. As part of this commitment we make the peer review history of every article we publish publicly available.

When an article is published we post the peer reviewers' comments and the authors' responses online. We also post the versions of the paper that were used during peer review. These are the versions that the peer review comments apply to.

The versions of the paper that follow are the versions that were submitted during the peer review process. They are not the versions of record or the final published versions. They should not be cited or distributed as the published version of this manuscript.

BMJ Open is an open access journal and the full, final, typeset and author-corrected version of record of the manuscript is available on our site with no access controls, subscription charges or pay-per-view fees (http://bmjopen.bmj.com).

If you have any questions on BMJ Open's open peer review process please email info.bmjopen@bmj.com

BMJ Open

Cohort Profile: The 1,000 Families Study, a UK multi-wave cohort investigating the wellbeing of families of children with intellectual disabilities.

Journal:	*BMJ Open*
Manuscript ID	bmjopen-2019-032919
Article Type:	Cohort profile
Date Submitted by the Author:	17-Jul-2019

Complete List of Authors:	Hastings, Richard; University of Warwick, Centre for Educational Development, Appraisal and Research (CEDAR); Monash University, Centre for Developmental Psychiatry and Psychology, Department of Psychiatry<br>Totsika, Vasiliki; University College London, Division of Psychiatry; University of Warwick, Centre for Educational Development, Appraisal and Research (CEDAR)<br>Hayden, Nikita; University of Warwick, Centre for Educational Development, Appraisal and Research (CEDAR)<br>Murray, Caitlin; University of Warwick, Centre for Educational Development, Appraisal and Research (CEDAR)<br>Jess, Mikeda; University of Warwick, Centre for Educational Development, Appraisal and Research (CEDAR)<br>Langley, Emma; University of Warwick, Centre for Educational Development, Appraisal and Research (CEDAR)<br>Margetson, Jane; University of Warwick, Centre for Educational Development, Appraisal and Research (CEDAR); Cerebra

Keywords:	Family Research, Intellectual Disability, Wellbeing, Longitudinal

SCHOLARONE™
Manuscripts

Cohort Profile: The 1,000 Families Study, a UK multi-wave cohort investigating the wellbeing of families of children with intellectual disabilities

Author names

Richard P. Hastings[1, 2], Vasiliki Totsika[3, 2, 1], Nikita K. Hayden[1], Caitlin A. Murray[1], Mikeda Jess[1], Emma Langley[1], & Jane Margetson[1, 4].

Email addresses: R.Hastings@warwick.ac.uk, V.Totsika@ucl.ac.uk, N.Hayden@warwick.ac.uk, C.Murray.7@warwick.ac.uk, M.Jess@warwick.ac.uk, E.Langley@warwick.ac.uk, J.Margetson@warwick.ac.uk

Corresponding author: Caitlin A. Murray (C.Murray.7@warwick.ac.uk, 024 76575866)

Author affiliations

[1] Centre for Educational Development, Appraisal and Research, University of Warwick, Coventry, UK

[2] Centre for Developmental Psychiatry and Psychology, Department of Psychiatry, Monash University, Melbourne, Australia

[3] Division of Psychiatry, University College London, London, UK

[4] Cerebra, Carmarthen, UK

Word count: 3338

For peer review only

ABSTRACT

Purpose: The 1,000 Families study is a large, UK-based, cohort of families of children with intellectual disabilities (ID). The study aims to describe and explore correlates of the wellbeing of families of children with ID, including parents and siblings, using cross-sectional and longitudinal data.

Participants: The study has recruited over 1,000 families of children with ID aged between 4 years and 15 years 11 months in the United Kingdom (N = 1189). Data were collected on family, parental and child wellbeing, as well as demographic information. Wave 1 data collection took place between November 2015 and January 2017, primarily through online questionnaires. Telephone interviews were also completed by 649 primary caregivers.

Findings to date: The mean age of the cohort was 9.03 years old. The cohort includes more boys (61.9%) than girls (27.0%; missing 11.1%). Parents reported that 45.6% (*n* = 542) of the children have autism. Most respondents were a female primary caregiver (84.8%), and 77.9% were the biological mother of the cohort child with ID. The largest ethnic group for primary caregivers was White British (78.5%), over half were married and living with their partner (53.3%), and 39.1% were educated to degree level.

Future plans: Wave 2 data collection is ongoing and the research team aims to continue following up these families in subsequent waves, subject to funding availability. Results will be used to inform policy and practice on family and child wellbeing in families of children with ID.

Keywords: Intellectual Disability, Family Research, Wellbeing, Longitudinal

For peer review only

ARTICLE SUMMARY

Strengths and limitations of this study

- A study of parental caregivers of children with ID to explore the wellbeing of children with ID, the wellbeing of parents and siblings, and the family system and relationships.
- The study includes over 1,000 families.
- Data will be collected longitudinally over a number of years, allowing investigation of family experiences over time.
- For the first two study waves, data are based on parental report only.
- The study may be biased towards those who have the time and motivation to participate.

INTRODUCTION

Intellectual Disability (ID) is a condition described in ICD-11 as a Disorder of Intellectual Development[1]. Consistent with contemporary definitions of this condition, ID emerges during the “developmental period” (usually taken to mean before age 18 years), and is characterised by low cognitive ability (IQ <70) and low levels of adaptive behaviour (such as communication, social skills, independence skills - also assessed using standardised tools). Prevalence studies internationally suggest that approximately 2-3% of children and adolescents have an ID[2-3].

For several decades, researchers throughout the world have asked questions about whether the health (especially mental health) of family members of children with ID is different to that in families that are not raising a child with ID. Meta-analyses and results from higher quality research designs (such as population-based national samples) have suggested that mothers of children with ID are about 1.5 times more likely than other mothers to experience depression[4], and similarly 1.4 times more likely than other mothers to have high

For peer review only

levels of psychological symptoms indicative of mental health problems[5]. Data from the UK Millennium Cohort Study (a population representative birth cohort) also show that fathers of young children with ID are twice as likely to score above the cut-off on a psychiatric disorder screen when compared to fathers of other young children[6] and older siblings of young children with ID are 1.5 times more likely to score in the "abnormal" range on a screen for behavioural and emotional problems when compared to other siblings of young children[7].

Sometimes explicitly, but often implicitly, researchers making these comparisons between families of children with ID and those without are drawing on a family systems perspective that has been applied to families of children with a variety of severe cognitive and developmental disabilities[8-11]. The core idea is that families are systems and so changes in one part of the family system, or in one family sub-system (e.g., spousal, parent-child, sibling-sibling), will affect other family members and/or other family sub-systems.

Families of children with ID face multiple challenges. For example, children are more likely to, by definition, have cognitive and adaptive skill deficits, and are 4-5 times more likely to have mental health problems compared to other children[12]. Families are also at increased risk for multiple social/economic disadvantage including poverty and negative life events[12]. The day-to-day burden of care for a child with ID is high even when compared with other carers (e.g., dementia family carers)[13], but access to supports and services for children with ID and their families is limited or fraught with negative experiences often described as "battling" against the system[14,15].

Given these challenges facing families of children with ID, we might expect multiple negative effects on (for example) the psychological adjustment of parents and siblings. Research findings are generally consistent with this prediction. From a systems perspective, we would also expect family sub-systems to be negatively affected. Again, research evidence supports this prediction. For example, using the population-based UK Millennium Cohort

For peer review only - http://bmjopen.bmj.com/site/about/guidelines.xhtml

Study, we showed that parents of young children with ID reported more conflict and lower levels of closeness in the relationship with their child compared to other parents of young children[16]. In addition, meta-analytic reviews suggest that parents of children with disabilities, including ID, may be more likely to separate/divorce and have lower levels of satisfaction with their partner relationship compared to other parents[17,18]. We developed the 1,000 Families Study to explore these issues further and to extend current knowledge across several domains.

Study aims

The main aims of the 1,000 Families Study are:

1. To describe the wellbeing of families of children with ID and to compare these data wherever possible with normative data and/or to national datasets (especially the UK Millennium Cohort Study). Family members include mothers (or primary parental caregivers), fathers (or secondary parental caregivers), and siblings.
2. To examine wellbeing differences between mothers and fathers in families of children with ID.
3. To explore correlates of maternal, paternal and sibling wellbeing and relationship quality, including: deprivation and socio-economic circumstances; other child, parent, sibling and family demographic factors; other family members' wellbeing, and the child with ID's behaviour problems and adaptive skills.
4. To explore correlates of the behavioural and emotional wellbeing of the child with ID, including: family deprivation; other child, parent, sibling and family demographic factors; other family members' wellbeing, the child with ID's adaptive skills, and parenting attitudes and behaviours.

COHORT DESCRIPTION

For peer review only

To date, we have conducted an online cross-sectional survey and telephone interviews of over 1,000 families of children with ID. Recruitment involved a multi-point contact method using websites, social media, advertisements in disability organisations' newsletters, and by approaching special schools and parent support organisations. To examine longitudinal associations, we are currently inviting these same families to participate in further data collection (Wave 2), which includes an online (or postal on request) survey but no telephone interview. To summarise the first and second waves of the 1,000 Families study:

Wave 1 data collection

The primary (and secondary if available) parental caregiver were asked to complete the same online survey: 1189 primary caregivers and 93 secondary caregivers completed the Wave 1 survey between November 2015 and January 2017. The primary caregiver was also invited take part in a telephone interview following the questionnaire to provide information about the adaptive skills and behaviours of their child with ID. The telephone interview was arranged at a time convenient to the primary caregiver and most often took around an hour. The telephone interviews were completed by 649 primary caregivers.

Wave 2 data collection

Participants for Wave 2 are the caregivers who indicated during Wave 1 that they were willing to be contacted again to be invited to participate in Wave 2. The telephone interview was replaced with an additional measure on adaptive skills in the online survey. Caregivers are first contacted 2 years and 9 months after they completed Wave 1, and are sent an initial email, two reminder emails, and then a final third reminder email which also asks if caregivers would no longer like to receive emails from the research team. Wave 2 data collection began in August 2018 and is anticipated to end in January 2021.

Study funding

For peer review only

The study is funded by Cerebra, a non-profit charity working with families with children with brain conditions. The project has also received funding from the Economic and Social Research Council Warwick Doctoral Training Centre, and the University of Warwick via PhD scholarship funding.

Study population

Inclusion criteria

1. For Wave 1, families with one or more children aged between 4 years and 15 years 11 months old who have an ID as reported by a parental caregiver. The child age range was initially (from November 2015) specified as 4 years to 11 years. In February 2017, an ethics amendment allowed us to expand recruitment to children aged up to 15 years and 11 months to meet families' interest in the study and to reach our sample target of over 1,000 families.
2. Families with at least one parental caregiver responding to the survey.
3. Families who live within the UK (i.e. England, Northern Ireland, Scotland or Wales).

Exclusion criteria

1. For Wave 1, families in which the child with ID lives outside of the family home on a full time basis (e.g., in a 52 week residential school placement).
2. Parental caregivers whose English literacy skills would not allow them to participate in an online survey and telephone interview.
3. Parental caregivers under 18 years old.

Measures

The study captured information on parental positivity, parental mental health, care burden/stress, life satisfaction, parent relationship, family satisfaction, sibling behaviour and emotional symptoms, sibling relationship quality, parenting, child behavioural and emotional wellbeing, child adaptive behaviour, sibling behavioural and emotional wellbeing and sibling

For peer review only

relationship quality. Table 1 summarises the key socio-demographic variables of the cohort, while Tables 2 and 3 summarise the remaining study measures, with number of missing items for each variable during Wave 1 indicated. The socio-economic and demographic variables of the cohort are described further in the findings section.

Patient and public involvement

The survey content and method was designed collaboratively with Cerebra, an organisation that supports and advocates for families of children with developmental disabilities, including ID. The final author (JM) participated in the design of the survey in her capacity as Cerebra staff member and PhD student at the University of Warwick. To date, the research team and Cerebra have collaborated to disseminate findings to families of children with ID through parent and practitioner research seminars Cerebra regularly hosts.

Ethics

The study is being conducted in accordance with the ethical principles of the British Psychological Society. Approval was obtained from the National Health Service (NHS) West Midlands – South Birmingham Research Ethics Committee prior to commencement of the study. Sponsor approval was also obtained from the University of Warwick. There are no significant risks for the families from taking part in this study, but as some of the questions included in the survey ask about potentially challenging issues, such as parental and child wellbeing and difficulties, signposting to various resources and helplines has been provided in the information sheet and at the end of the survey for caregivers.

All participant information is stored in line with the requirements of the Data Protection Act 1998. While this study was approved and began before changes to the General Data Protection Regulation (GDPR) in 2018, data collection and procedures in Wave 2 are in line with GDPR requirements. Caregivers are informed through information sheets and

For peer review only

**Table 1.** Socio-economic and demographic information for Wave 1 Cerebra 1,000 Families sample	
**Child**	
Child age range (years)	4.00-15.92
Child age: mean (SD) (years)	9.03 (3.05)
Missing age n	187 (15.7%)
Child gender:	
n of males	736 (61.9%)
n of females	321 (27.0%)
Missing gender n	132 (11.1%)
Parental caregiver reported ID Level	
Severe to profound ID	503 (42.3%)
Mild to moderate ID	503 (42.3%)
Missing information	183 (15.4%)
Parental caregiver reported Autism diagnosis of child	542 (45.6%)
Down syndrome	162 (13.6%)
Type of School:	
n of children attending special school	465 (39.1%)
n of children attending mainstream school	339 (28.3%)
n of children attending mainstream school in either a special unit or special educational needs provision	151 (12.7%)
Missing information	178 (15.0%)
**Primary Caregiver and Family**	
Female Primary Caregiver	1008 (84.8%)
Primary Caregiver Relationship to Child	
Biological Mother	926 (77.9%)
Adoptive Mother	53 (4.5%)
Biological Father	46 (3.9%)
Grandmother	13 (1.1%)
Missing information	131 (11.0%)
Primary Caregiver Employment status:	

For peer review only

1

In a job working for an employer	392 (33.0%)
Looking after home and family	388 (32.6%)
Self-employed	103 (8.7%)
Doing something else	101 (8.5%)
Missing information	131 (11.0%)
Primary caregiver education level:	
Degree level	465 (39.1%)
Higher education but below degree level	255 (21.4%)
A/AS Levels or equivalent	105 (8.8%)
5 or more GCSEs at A-C or equivalent*	74 (6.2%)
Some GCSEs passes or equivalent	95 (7.8%)
No qualifications	13 (1.1%)
Missing information	184 (15.5%)
Primary caregiver relationship status:	
Married and living with spouse/civil partner	634 (53.3%)
Divorced/separated/single/widowed/not currently living with partner	226 (19.0%)
Living with partner	141 (11.9%)
Missing information	188 (15.8%)
Primary Caregiver Ethnicity:	
White British	933 (78.5%)
White other	40 (3.4%)
Asian/Asian British	26 (2.3%)
Black (African/Caribbean/Black British)	15 (1.3%)
Remaining ethnic groups (Mixed / multiple ethnicity, Arabic, Any other ethnic background etc.)	23 (2.0%)
Missing information	137 (11.5%)
Family living in most deprived 10% of neighbourhoods based on indices of multiple deprivation (IMD)	90 (7.6%)
Missing IMD information	36 (3.0%)

For peer review only

Table 2. Parent and family measures used within the Cerebra 1,000 Families Study

Domain	Measure	Scoring	Measured at	Wave 1 Measure completeness
Parental positivity	Positive gains scale [Jess et al., Measurement invariance of the Positive Gains Scale in families of children with and without disabilities; under review[1]]<br>7 items<br>Perceived benefits for parent (5 items)<br>What family has gained (2 items)	5-point scale from 0 (*strongly agree*) to 4 (*strongly disagree*). Original scale had a lower score indicating higher positive gains. The scoring was reversed so that a higher score indicates higher positive gains.	T1, T2	Complete *n* = 1007<br>Missing *n* = 182
Parent mental health	Kessler 6 (K6)[33]<br>6 items	5-point Likert scale asks caregivers how often they've experienced six symptoms over the last 30 days from 0 (*none of the time*) to 4 (*all of the time*). Total score is calculated by summing the responses. A score of 13 and above indicates serious mental illness[34].	T1, T2	Complete *n* = 1003<br>Missing *n* = 186
Carer burden/stress	'Impact of care-giving on carer'<br>Survey of Informal Carers in Households 2009/2010[35]<br>7 items	Items ask individuals whether certain aspects of their lives have been affected by caring for another (*Yes* or *No*). Totals are generated for the responses, and higher scores indicate higher carer burden/stress.	T1, T2	Complete *n* = 965<br>Missing *n* = 224
Life satisfaction	Life satisfaction scale[36]<br>1 item	Single item measure that asks caregivers to rate their general life satisfaction from 1 (*completely dissatisfied*) to 10 (*completely satisfied*). Scores 6-10 indicate that respondents are 'satisfied overall'. Scores 4 and below indicate that participants are 'dissatisfied overall'.	T1, T2	Complete *n* = 1001<br>Missing *n* = 188
Parent relationship – parent's perception of relationship with their partner (if applicable)	Disagreement over issues related to child[37]<br>1 item	This item asks caregivers to report on how often they disagree over issues relating to their child on a 6-point Likert scale (*Never* to *More than once a day*). There is also a response for 'Can't say'.	T1, T2	Complete *n* = 773<br>No live-in partner *n* = 226<br>Missing *n* = 190
	Happiness of relationship scale[37]<br>1 item	Caregivers are asked to rate how happy they are in their relationship from 1 (*very unhappy*) to 7 (*very happy*). There is also a response for 'Can't say'.	T1, T2	Complete *n* = 768<br>No live-in partner *n* = 226<br>Missing *n* = 195
Family functioning	Family APGAR scale[38]<br>5 items	Respondents are required to rate the frequency of feeling satisfied with each parameter on a 3-point Likert scale, from 0	T1, T2	Complete *n* = 996<br>Missing *n* = 193

[1] cf. Jess M, Bailey T, Pit-ten Cate, IM, *et al.* Measurement invariance of the Positive Gains Scale in families of children with and without disabilities. 2019. [submitted for review]

	Adaptability, Partnership, Growth, Affection and Resolve (APGAR)	(*hardly ever) to* 2 (*almost always*). The scale is scored by summing the 5 items. A higher score indicates a greater degree of satisfaction with family functioning (8-10 = *highly functional*, 4-7 = *moderately dysfunctional*, 0-3 = *dysfunctional*).		
Sibling behavioural and emotional wellbeing	Strengths and Difficulties Questionnaire (SDQ) (Parent questionnaire)[39]<br>25 items<br><br>Subscales: Emotional problems, Conduct problems, Hyperactivity/inattention, Peer relationship problems, Prosocial behaviour	Caregivers are asked to assess the extent to which each statement applies to the child based on the last 6 months using a 3-point rating scale (0 = *not true* to 2 = *certainly true*). The items are divided into 5 subscales. A total difficulties score can be obtained by summing the first four sub-scale scores: *close to average* (0-13), *slightly raised* (14-16), *high* (17-19), and *very high* (20-40).	T1, T2	Complete *n* = 606<br>No sibling *n* = 361<br>Missing *n* = 222
Sibling relationship	Sibling Relationship Questionnaire (SRQ) (Parent questionnaire) (revised)[40]<br>10 items<br>Adapted version using key items from two subscales of SRQ – SF:<br>Warmth and closeness (6 items)<br>Conflict (4 items)	Caregivers are asked about the sibling relationship on a 5-point Likert scale from 1 (*very much*) to 5 (*extremely much*). These items can then be used to derive measures that encapsulate positive or negative aspects of the sibling relationship.	T1, T2	SRQ warmth:<br>Complete *n* = 598<br>No sibling *n* = 361<br>Missing *n* = 230<br>SRQ conflict:<br>Complete *n* = 602<br>No sibling *n* = 361<br>Missing *n* = 226
Parenting attitudes and behaviours	Child-parent relationship scale (CPRS)[41]<br>15 items<br>Conflict (8 items)<br>Closeness (7 items)	The caregiver states their feelings and beliefs about their relationship with their child and about the child's behaviour towards them, by responding to statements on a 5-point Likert scale (1 = *Definitely does not apply* to 5 = *Definitely applies*). CPRS generates a separate score for the individual conflicts and closeness constructs by summing the scores in each subscale	T1, T2	Closeness:<br>Complete *n* = 981<br>Missing *n* = 208<br>Conflict:<br>Complete *n* = 986<br>Missing *n* = 203
	Alabama Parenting questionnaire (Parent form)[42]<br>12 items<br>Positive parenting (6 items) Inconsistent discipline (6 items)	Parents/caregivers are asked about their parenting behaviours on a five-point Likert scale (1 = *Never* to 5 = *Always*). The items in each scale are summed to obtain two scale scores.	T1, T2	Positive parenting:<br>Complete *n* = 974<br>Missing *n* = 215<br>Inconsistent discipline:<br>Complete *n* = 963<br>Missing *n* = 226
	Parent activities/involvement index<br>5 items	This questionnaire was constructed for the purposes of this study. Some of the questions have been used in national UK surveys such as the Millennium Cohort Study, and where relevant, items were adapted to be more appropriate for families of children with ID. The questions ask about parent-child interactions for activities and parental involvement with their	T1<br>*Removed from T2 due to parental feedback.*	Complete *n* = 979<br>Missing *n* = 210

		child. Answers are provided on a 5-point Likert scale (from 1 = *Not at all* to 5 = *Every day*).		

Table 3. Child measures used within the Cerebra 1,000 Families Study

Domain	Measure	Scoring	Measured at	Wave 1 Measure completeness
Child with ID behavioural and emotional wellbeing	Strengths and Difficulties Questionnaire (SDQ) (Parent questionnaire)[39]<br>25 items<br>Subscales: Emotional problems, Conduct problems, Hyperactivity/inattention, Peer relationship problems, Prosocial behaviour	Caregivers are asked to assess the extent to which each statement applies to the child based on the last 6 months using a 3-point rating scale (0 = *not true* to 2 = *certainly true*). The items are divided into 5 subscales. A total difficulties score can be obtained by adding the first four sub-scale scores and categorising them into a four band system: *close to average* (0-13), *slightly raised* (14-16), *high* (17-19), and *very high* (20-40).	T1, T2	Complete *n* = 990<br>Missing *n* = 199
Behavioural and emotional problems of the child with ID	Development behaviour checklist (parent version) (DBC-P)[43]<br>96 items<br>Telephone interview<br>Subscales: Disruptive/Anti-social Behaviour, Self-absorbed, Communication Disturbance, Anxiety, Social Relating	Items are scored on a 0, 1 or 2 rating scale (0 = *Not true as far as you know*, 1 = *Somewhat or sometimes true*, 2 = *Very true or often true*). The Total Behaviour Problem Score (TBPS) is an overall measure of emotional and behavioural problems, and can detect clinically significant levels of overall emotional and behavioural disturbance (indicated by a score of 46 or greater).	T1	Complete *n* = 649<br>Did not consent to telephone interview *n* = 199<br>Missing *n* = 341
Adaptive behaviour skills of the child with ID	Vineland adaptive behaviour scales – second edition (parent interview form) (VABS- II)[44]<br>30-40 minute telephone interview<br><br>Communication Skills<br>Daily Living Skills<br>Socialisation Skills	An overall composite score, and domain scores, can be derived with reference to age during typical development during which children can perform the task items. Four standardised scores in total were derived for the present research: Adaptive Behaviour Composite, Communication Skills, Daily Living Skills, and Socialisation Skills. Age Equivalency scores were also calculated.	T1	Complete *n* = 645<br>Did not consent to telephone interview *n* = 199<br>Missing *n* = 345
Adaptive behaviour skills of the child with ID	The GO4KIDDS Brief Adaptive Scale[45]<br>9 items	Items cover communication, self-help skills, social interaction, and support needs. Each item is rated on a 5-point scale where higher scores indicate greater skill level and greater independence. These item scores are summed to get an overall Adaptive Behaviour	T2	Not yet known

	score. We have amended the original GO4KIDDS to include an additional item asking about alternative methods of communication following feedback from caregivers.	Replaced the VABS-II interview

For peer review only

consent forms on what data are collected, why this is necessary, and how these data are stored. All aspects of the study are conducted on the basis of explicit written consent to take part in each stage of the study. All research data are stored electronically and password protected on firewalled University computers with access limited to the research team. The password and access to the research data, including participant information, is restricted to the Chief Investigator and the research team.

For Wave 2 of the data collection, consent is collected as it was in Wave 1 and participants are asked again if they would be willing to be contacted for any future research. At Wave 2, a £10 gift voucher was introduced as a Thank You to caregivers who complete the survey. To mitigate against potential biases arising from the monetary incentive, it is made clear in the information sheet that caregivers do not have to answer any questions that make them feel uncomfortable and they will still receive the voucher.

Dissemination

Throughout the study, results will be published in peer-reviewed academic journals. Presentations of study findings will be made at research conferences and seminars. The charity Cerebra and other stakeholders will be involved in methods of dissemination for reaching the wider community, particularly families of children with ID. Caregivers who took part in the study, and have consented, receive updates about the study through a newsletter and social media, which the public can also access. This allows findings to be summarised and presented in an accessible format in addition to any academic papers or presentations.

FINDINGS TO DATE

The socio-economic and demographic characteristics of the cohort are presented in Table 1. All data reported in this table were reported by the primary caregiver of the child with ID. These were usually the biological mother of the child with ID (77.95%) or another

For peer review only

female relative (84.8% of the primary caregivers were female). Cohort children with ID were aged between four years and 15 years and nine months, with a mean age of 9.03 (SD = 3.05). There were more boys than girls (61.9% were male; 27.0% were female; 11.1% missing). In terms of ID severity, 42.3% were reported to have a severe to profound ID, and 42.3% a mild to moderate ID (15.4% missing). Additional diagnoses were also reported, with 45.6% of the sample reporting an additional autism diagnosis, and 13.6% having a Down syndrome diagnosis.

Comparison with indicators from the 2011 census of England and Wales[19] indicate lower participation of families identifying as Black, Asian or Minority Ethnic (5.6% of the 1,000 Families sample compared to 13% of the population of England and Wales), but expected numbers of families identifying as White British (78.5% of the 1,000 Families sample, compared to 80.5% in the 2011 Census) (see Table 1 for full ethnicity information on the 1,000 Families). In terms of educational qualifications, the survey sample were more highly educated (39.1% of the primary caregivers in the 1,000 Families were educated to degree level, in comparison to 27% in the England and Wales 2011 Census)[19]. Whilst 23% of the population in England and Wales had no educational qualifications, only 1.1% of the 1,000 Families sample reported having no educational qualifications. These comparisons to the census data should be viewed with caution as the census data about qualifications include the full age-range of adults living in England and Wales in 2011, whereas our sample of primary caregivers disproportionately includes women of childrearing age across all four countries of the UK. Policy changes leading to the expansion of UK higher education in recent decades means we would expect that the primary caregivers in our sample were more likely to have completed degrees than were previous generations. Furthermore, the 2011 census data included people aged 16-18 and this group were less likely to have any qualifications yet, although they may have been in education. In terms of socio-economic

For peer review only

indicators, 7.6% of the families in the 1,000 Families sample were living in the most deprived areas in their respective country based on the indices of multiple deprivation for England, Scotland, Wales and Northern Ireland [20-23]. These indices offer a measure of relative deprivation at a local scale by combining information on income poverty, education, unemployment health, crime, barriers and access to services, housing, and physical environment for each small area and then rank-ordering areas within each country.

Future directions

The data will be analysed in cross-sectional and longitudinal studies. Where possible, data will be compared with data from normative or population-representative samples. Correlation and regression approaches will be used to explore factors associated with the wellbeing of mothers, fathers, siblings, and the child with ID. Where appropriate, models will account for the nested nature of data within families.

STRENGTHS AND LIMITATIONS

This study is, as far as we are aware, the largest cohort of families with a child with ID in the UK and will make an important contribution to the research on the wellbeing of children with ID and their families. Particularly important is the longitudinal design which currently includes a second wave of data collection that is underway. Selection of measures allows for some comparison with population-representative samples in the UK (e.g. the Millennium Cohort Study[24]). Results will inform parents, clinicians, and educational professionals on factors related to the wellbeing of children with ID, their parents, and their siblings.

Designing this research study via a questionnaire and telephone interview allowed us to recruit a large sample from across the UK. However, this precluded an independent ascertainment of child ID, and/or associated developmental conditions (e.g., autism spectrum disorders). Instead, information on the child’s diagnoses is based solely on parental report.

For peer review only

The implication of this limitation is that the survey cannot verify the extent of clinical diagnoses available for each child with ID. Where research evidence has examined the validity of parental reports, it has suggested that parents' reports correspond well to clinical diagnoses available[25,26] and that parental reports on children's developmental outcomes are accurate[27].

Although the sample size recruited at Wave 1 is large (>1,000 families), the sampling design (convenience sample recruited through social media and ID-related organisations) may have resulted in sampling bias, where families of children with ID in contact with charitable organisations and/or access to social media may be more likely to be included. Although not fully representative of the UK, the findings discussed previously indicate that the 1,000 Families sample includes elements of diversity that have the potential to provide insights into families with a child with ID from various backgrounds.

An additional limitation may have been related to the time requirements for data completion at Wave 1 (survey and telephone interview). Families who completed both elements of the survey may have had more time available to give to research. In addition, fewer primary caregivers with higher levels of poverty (55.4%) completed the telephone interview than those with lower levels of poverty (69.4%). To identify which families live in poverty, we used four variables including: subjective poverty, hardship, income poverty, and neighbourhood deprivation. Those with no or one indicator of poverty were identified as having lower levels of poverty, and those with two or more indicators of poverty were identified as having higher levels of poverty. In terms of qualifications, 30.8% of those with no qualifications completed a telephone interview compared to 67.3% of those with a degree. Primary caregivers who were single parents provided a telephone interview in 54.0% of cases, compared to primary caregivers from two-parent households, who provided a telephone interview in 65.4% of cases.

For peer review only

Children of caregivers who did not take part in the telephone interview had higher total SDQ scores with a mean score of 18.3 for the children whose primary caregivers did not take part in the telephone interview compared to a mean score of 16.8 for those who did. The child with ID having multiple diagnoses, however, did not decrease the chances that a primary caregiver would complete a telephone interview. For example, those children with ID whose primary caregivers completed a telephone interview were more likely to have a coexisting physical health problem (such as epilepsy, mobility problems, hearing or visual problems). The children whose primary caregivers completed a telephone interview had a mean number of health problems of 1.70 compared to 1.06 for the children with ID whose primary caregivers did not complete a telephone interview. Additionally, the children whose primary caregivers completed a telephone interview were more likely to have a greater number of diagnostic labels, with a mean number of 2.18 compared to 1.45 for the children with ID whose primary caregivers did not complete a telephone interview.

Last, although 779 of the primary caregivers in the sample indicated they were married and/or living with a partner, only 93 secondary caregivers participated in the survey. Overall low rates of participation of fathers or secondary caregivers in family research is a well-described phenomenon that may relate to role allocation between parents regarding child care, working commitments, and primary responsibility for contact with disability and educational services[28-32]. The 1,000 Families study directly recruited and encouraged fathers to take part in the study, but it is likely that a recruitment strategy primarily focused on the family was not specific enough to encourage greater participation from fathers and secondary caregivers. There is a need for further investigation on why other family members, particularly fathers, were not as likely to take part, and whether there are recruitment strategies which are more successful to reach fathers and secondary caregivers, as some

For peer review only

research has highlighted the importance of inviting fathers directly and emphasizing the importance of their participation[32, 28].

COLLABORATION

Initial data analyses and publications will be generated by researchers at CEDAR, University of Warwick as part of the 1,000 Families study funded by Cerebra. However, the research team welcome researchers interested in future collaboration to contact the corresponding author with their expression of interest. Access and analysis of the data is currently only possible via the University of Warwick, due to the ethical approvals in place.

Acknowledgements We would like to thank all the families that participated in this study. We acknowledge the support from Cerebra in funding, developing and recruiting to this study; and the ESRC Doctoral Training Centre at the University of Warwick for funding support in the form of PhD studentships. We acknowledge the contributions of our study administrators, Alison Baker and Diana Smith. Various researchers assisted in conducting telephone interviews, alongside the authors, to collect data for Wave 1 of this study and we acknowledge their support: Latoya Clarke, Farah Elahi, Gemma Gray, Liz Halstead, Sasha Mandair, Louise Rixon, Suzi Scott and Ruth Thomas.

Author Contributions RH and VT conceptualised and co-designed the study, wrote the study protocol, contributed to the drafting of the manuscript for publication, reviewed and revised the manuscript, and read and approved the final manuscript. NH and CM contributed to the design of the study, contributed to the drafting of the manuscript for publication, reviewed and revised the manuscript, and read and approved the final manuscript. MJ, EL and JM contributed to the design of the study, contributed to the original protocol, and read and approved the final manuscript.

For peer review only

Funding statement The authors acknowledge the funding support of Cerebra, UK, the Economic and Social Research Council Warwick Doctoral Training Centre, and the University of Warwick.

Competing Interests Statement None declared.

Patient consent Parental/guardian consent obtained.

Data Availability Statement Data from this study are not available for sharing due to ethical approval requirements. Researchers interested in collaboration should contact the corresponding author with their expression of interest.

Ethics approval: Full ethical approval was granted for this study from the UK National Health Service (NHS) West Midlands – South Birmingham Research Ethics Committee: REC reference number: 15/WM/0267 (11th September 2015). An amendment was granted to extend the age range (7th March 2017). An amendment was granted for changes made to Wave 2 (8th February 2018) and the ethics committee was informed of further minor changes made to the documents for Wave 2 (27th February 2018). Sponsor approval was also obtained from the University of Warwick.

For peer review only

REFERENCES

1. Salvador-Carulla L, Reed GM, Vaez-Azizi, LM, *et al.* Intellectual developmental disorders: towards a new name, definition and framework for "mental retardation/intellectual disability" in ICD-11. *World Psychiatry* 2011;10(3):175-180. doi:10.1002/j.2051-5545.2011.tb00045.x

2. Maulik PK, Mascarenhas MN, Mathers CD, *et al.* Prevalence of intellectual disability: A meta-analysis of population-based studies *Res Dev Disabil* 2011;32:419-436. doi:10.1016/j.ridd.2010.12.018

3. Totsika V, Hastings RP, Emerson E, *et al.* A population-based investigation of behavioural and emotional problems and maternal mental health: Associations with autism and intellectual disability. *J Child Psychol Psychiatry* 2011;52:91-99. doi:10.1111/j.1469-7610.2010.02295.x

4. Singer GH. Meta-analysis of comparative studies of depression in mothers of children with and without developmental disabilities. *Am J Ment Retard* 2006;111:155-169. doi:10.1352/0895-8017(2006)111[155:MOCSOD]2.0.CO;2

5. Emerson E. Mothers of children and adolescents with intellectual disability: Social and economic situation, mental health status, and the self-assessed social and psychological impact of the child's difficulties. *J Intellect Disabil Res* 2003;47:385-399. doi:10.1046/j.1365-2788.2003.00498.x

6. Emerson E, McCulloch A, Graham H, *et al.* Socioeconomic circumstances and risk of psychiatric disorders among parents of children with early cognitive delay. *Am J Intellect Dev Disabil* 2010;115:30-42. doi:10.1352/1944-7558-115.1.30

7. Hayden NK, Hastings RP, Totsika V, *et al.* A Population-Based Study of the Behavioral and Emotional Adjustment of Older Siblings of Children with and without Intellectual

For peer review only

Disability. *J Abnorm Child Psychol* Published Online First: 4 February 2019. doi:10.1007/s10802-018-00510-5

8. Cridland EK, Jones SC, Magee CA, *et al.* Family-focused autism spectrum disorder research: a review of the utility of family systems approaches. *Autism* 2014;18:213-222. doi:10.1177/1362361312472261

9. Kovshoff H, Cebula K, Tsai H-WJ, *et al.* Siblings of children with autism: The Siblings Embedded Systems Framework. *Curr Dev Disord Rep* 2017;4(2):37-45. doi:10.1007/s40474-017-0110-5

10. Seligman M, Darling RB. *Ordinary families, special children.* 3rd ed. New York: Guilford Press, 2007.

11. Trivette CM, Dunst CJ, Hamby DW. Influences of family-systems intervention practices on parent-child interactions and child development. *Topics Early Child Spec Educ* 2010;30:3-19. doi:10.1177/0271121410364250

12. Emerson E, Hatton C. Mental health of children and adolescents with intellectual disabilities in Britain. *Br J Psychiatry* 2007;191:493–499. doi:10.1192/bjp.bp.107.038729

13. Totsika V, Hastings RP, Vagenas D. Informal caregivers of people with an intellectual disability in England: Health, quality of life and impact of caring. *Health Soc Care Community* 2017:25:951-961. doi:10.1111/hsc.12393

14. Griffith GM, Hastings RP. “He’s hard work, but he’s worth it.” The experience of caregivers of individuals with intellectual disabilities and challenging behaviour: A meta-synthesis of qualitative research. *J Appl Res Intellect Disabil* 2014;27:401-419. doi:10.1111/jar.12073

15. Toms G, Totsika V, Hastings RP, *et al.* Access to services by children with intellectual disability and mental health problems: Population-based evidence from the UK. *J Intellect Dev Disabil* 2015;40:239-247. doi:10.3109/13668250.2015.1045460

For peer review only

16. Totsika V, Hastings RP, Vagenas D, *et al.* Parenting and the behavior problems of young children with an intellectual disability: Concurrent and longitudinal relationships in a population-based study. *Am J Intellect Dev Disabil* 2014;119:422-435. doi:10.1352/1944-7558-119.5.422

17. Risdal D, Singer GHS. Marital Adjustment in Parents of Children with Disabilities: A Historical Review and Meta-Analysis. *Res Pract Persons Severe Disabl* 2004;29:95-103. doi:10.2511/rpsd.29.2.95

18. Saini M, Stoddart KP, Gibson M, *et al.* Couple relationships among parents of children and adolescents with Autism Spectrum Disorder: Findings from a scoping review of the literature. *Res Autism Spectr Disord* 2015;17:142-157. doi:10.1016/j.rasd.2015.06.014

19. Ethnicity in the UK. *GOV.UK website, Ethnicity facts and figures* 2019. https://www.ethnicity -facts-figures.service.gov.uk/ethnicity-in-the-uk. Accessed 9th March 2019.

20. Gill B. *The English Indices of Deprivation 2015: Statistical Release*. London: Department for Communities and Local Government, 2015. https://www.gov.uk/government /statistics/english-indices-of-deprivation-2015. Accessed 16th June 2019.

21. *Introducing the Scottish Index of Multiple Deprivation 2016.* Edinburgh: APS Group Scotland for The Scottish Government, 2016. https://www2.gov.scot.Resource/ 0050/00504809.pdf Accessed 16th June 2019.

22. *Welsh Index of Multiple Deprivation 2014: Executive summary revised.* Knowledge and Analytical Services, Welsh Government, 2015. https://gweddill.gov.wales/docs/statistics /2015/150812-wimd-2014-summary-revised-en.pdf. Accessed 16th June 2019.

23. *Northern Ireland Multiple Deprivation Measures 2017: Technical Report.* Northern Ireland Statistics and Research Agency, 2017. https://www.nisra.gov.uk/sites/

For peer review only

nisra.gov.uk/files/publications/NIMDM%202017_Technical%20Report.pdf. Accessed 16th June 2019.

24. University of London, Institute of Education, Centre for Longitudinal Studies.. Millennium Cohort Study: Fourth Survey, 2008. [data collection]. 7th Edition. UK Data Service, 2017. SN: 6411. doi:10.5255/UKDA-SN-6411-7

25. Daniels AM, Rosenberg RE, Anderson C, *et al.* Verification of parent-report of child autism spectrum disorder diagnosis to a web-based autism registry. *J Autism Dev Disord* 2012;42(2):257-265. doi:10.1007/s10803-011-1236-7

26. Lee H, Marvin AR, Watson T, *et al.* Accuracy of Phenotyping of Autistic Children Based on Internet Implemented Parent Report. *Am J Med Genet **B** Neuropsychiatr Genet* 2010;0(6):1119–1126. doi:10.1002/ajmg.b.31103

27. Charman T, Howlin P, Berry B, *et al.* Measuring developmental progress of children with autism spectrum disorder on school entry using parent report. *Autism* 2004;8(1):89-100. doi:10.1177/1362361304040641

28. Costigan, CL, Cox, MJ. Fathers' participation in family research: is there a self-selection bias? *J Fam Psychol* 2001;15(4):706-720.

29. Cassano M, Adrian M, Veits G, *et al.* The inclusion of fathers in the empirical investigation of child psychopathology: an update. *J Clin Child Adolesc Psychol* 2006;35(4):583-589. doi:10.1207/s15374424jccp3504_10

30. Mitchell SJ, See HM, Tarkow AKH, *et al.* Conducting studies with fathers: Challenges and opportunities. *Appl Dev Sci* 2007;11(4):239–244. doi:10.1080/10888690701762159

31. Doyle O, Weller BE, Daniel SS, *et al.* Overcoming Barriers to Fathers' Participation in Clinically Relevant Research: Recommendations from the Field, *Social Work Research* 2016;40(4):260–264. doi:10.1093/swr/svw015

For peer review only

32. Phares V, Lopez E, Fields S, *et al.* Are fathers involved in pediatric psychology research and treatment? *J Pediatr Psychol* 2005;30(8):631-643. DOI: 10.1093/jpepsy/jsi050

33. Kessler RC, Andrews G, Colpe LJ, *et al.* Short screening scales to monitor population prevalences and trends in non-specific psychological distress. *Psychol Med* 2002;32:959-976. doi:10.1017/S0033291702006074

34. Kessler RC, Barker PR, Colpe LJ*, et al.* Screening for serious mental health difficulties in the general population. *Arch Gen Psychiatry* 2003;60:184-189 doi:10.1001/archpsyc.60.2.184

35. The NHS Information Centre, Social Care team. 451845 Survey of Informal Carers in Households 2009/10 Mainstage questionnaire [Internet]. NHS Information Centre; Leeds, 2010. p. 24. Available from: https://files.digital.nhs.uk/publicationimport/pub02xxx/pub02200/surv-care-house-care-ques-eng-2009-2010-apx2.pdf

36. Department for Environment, Food and Rural Affairs (DEFRA). Life satisfaction and other measures of wellbeing in England, 2007-2011 [Internet]. DEFRA; London, 2011. Available from: https://webarchive.nationalarchives.gov.uk/20130123225714/http://www.defra.gov.uk/statistics/files/Statistical-Release-13-April-2011-wellbeing.pdf

37. University of London, Institute of Education, Centre for Longitudinal Studies. Millennium Cohort Study Second Survey, 2003-2005: CAPI Questionnaire Documentation. [Internet]. Centre for Longitudinal Studies; London, 2007. p. 96-97. Available from: http://doc.ukdataservice.ac.uk/doc/5350/mrdoc/pdf/mcs2_capi_questionnaire_documentation_november_2007.pdf

38. Smilkstein G. The Family APGAR: A proposal for a family function test and its use by physicians. *The Journal of Family Practice* 1978;6:1231-1239.

For peer review only

39. Goodman R. The Strengths and Difficulties Questionnaire: A research note. *J Child Psychol Psychiatry* 1997;38:581-586. doi:10.1111/j.1469-7610.1997.tb01545.x

40. Furman W, Buhrmester D. Children's perceptions of the qualities of sibling relationships. *Child Development* 1998;56:448-461. doi:10.2307/1129733

41. Pianta RC. *Child-Parent Relationship Scale*. Charlottesville, VA: University of Virginia, 1995.

42. Frick PJ. The Alabama Parenting Questionnaire. Unpublished rating scale, University of Alabama, 1991.

43. Einfeld SL, Tonge BJ. *Manual for the Developmental Behaviour Checklist: Primary Carer Version (DBC-P) & Teacher Version (DBC-T) (2nd ed.)*. Clayton, Melbourne: Monash University Centre for Developmental Psychiatry and Psychology, 2002.

44. Sparrow SS, Cicchetti DV, Saulnier CA. *Vineland Adaptive Behavior Scales, Survey Forms Manual*. 3rd ed. Circle Pines, MN: AGS Publishing, 2016.

45. Perry A, Taheri A, Ting V, *et al.* The GO4KIDDS Brief Adaptive Scale. *J Appl Res Intellect Disabil* 2015;28:594-597. doi:10.1111/jar.12143

For peer review only

BMJ Open

Cohort Profile: The 1,000 Families Study, a UK multi-wave cohort investigating the wellbeing of families of children with intellectual disabilities.

Journal:	*BMJ Open*
Manuscript ID	bmjopen-2019-032919.R1
Article Type:	Cohort profile
Date Submitted by the Author:	28-Nov-2019
Complete List of Authors:	Hastings, Richard; University of Warwick, Centre for Educational Development, Appraisal and Research (CEDAR); Monash University, Centre for Developmental Psychiatry and Psychology, Department of Psychiatry<br>Totsika, Vasiliki; University College London, Division of Psychiatry; University of Warwick, Centre for Educational Development, Appraisal and Research (CEDAR)<br>Hayden, Nikita; University of Warwick, Centre for Educational Development, Appraisal and Research (CEDAR)<br>Murray, Caitlin; University of Warwick, Centre for Educational Development, Appraisal and Research (CEDAR)<br>Jess, Mikeda; University of Warwick, Centre for Educational Development, Appraisal and Research (CEDAR)<br>Langley, Emma; University of Warwick, Centre for Educational Development, Appraisal and Research (CEDAR)<br>Margetson, Jane; University of Warwick, Centre for Educational Development, Appraisal and Research (CEDAR); Cerebra
**Primary Subject Heading**:	Mental health
Secondary Subject Heading:	Public health, Paediatrics
Keywords:	Family Research, Intellectual Disability, Wellbeing, Longitudinal

SCHOLARONE™ Manuscripts

I, the Submitting Author has the right to grant and does grant on behalf of all authors of the Work (as defined in the below author licence), an exclusive licence and/or a non-exclusive licence for contributions from authors who are: i) UK Crown employees; ii) where BMJ has agreed a CC-BY licence shall apply, and/or iii) in accordance with the terms applicable for US Federal Government officers or employees acting as part of their official duties; on a worldwide, perpetual, irrevocable, royalty-free basis to BMJ Publishing Group Ltd ("BMJ") its licensees and where the relevant Journal is co-owned by BMJ to the co-owners of the Journal, to publish the Work in this journal and any other BMJ products and to exploit all rights, as set out in our licence.

The Submitting Author accepts and understands that any supply made under these terms is made by BMJ to the Submitting Author unless you are acting as an employee on behalf of your employer or a postgraduate student of an affiliated institution which is paying any applicable article publishing charge ("APC") for Open Access articles. Where the Submitting Author wishes to make the Work available on an Open Access basis (and intends to pay the relevant APC), the terms of reuse of such Open Access shall be governed by a Creative Commons licence – details of these licences and which Creative Commons licence will apply to this Work are set out in our licence referred to above.

Other than as permitted in any relevant BMJ Author's Self Archiving Policies, I confirm this Work has not been accepted for publication elsewhere, is not being considered for publication elsewhere and does not duplicate material already published. I confirm all authors consent to publication of this Work and authorise the granting of this licence.

For peer review only

Cohort Profile: The 1,000 Families Study, a UK multi-wave cohort investigating the wellbeing of families of children with intellectual disabilities

Author names

Richard P. Hastings[1, 2], Vasiliki Totsika[3, 2, 1], Nikita K. Hayden[1], Caitlin A. Murray[1], Mikeda Jess[1], Emma Langley[1], & Jane Margetson[1, 4].

Email addresses: R.Hastings@warwick.ac.uk, V.Totsika@ucl.ac.uk, N.Hayden@warwick.ac.uk, C.Murray.7@warwick.ac.uk, M.Jess@warwick.ac.uk, E.Langley@warwick.ac.uk, J.Margetson@warwick.ac.uk

Corresponding author: Caitlin A. Murray (C.Murray.7@warwick.ac.uk, 024 76575866)

Author affiliations

[1] Centre for Educational Development, Appraisal and Research, University of Warwick, Coventry, UK

[2] Centre for Developmental Psychiatry and Psychology, Department of Psychiatry, Monash University, Melbourne, Australia

[3] Division of Psychiatry, University College London, London, UK

[4] Cerebra, Carmarthen, UK

Word count: 3930

For peer review only

ABSTRACT

Purpose: The 1,000 Families study is a large, UK-based, cohort of families of children with intellectual disabilities (ID). The main use of the cohort data will be to describe and explore correlates of the wellbeing of families of children with ID, including parents and siblings, using cross-sectional and (eventually) longitudinal analyses.

Participants: Over 1,000 families of UK children with ID aged between 4 years and 15 years 11 months (total N = 1,184) have been recruited. The mean age of the cohort was 9.01 years old. The cohort includes more boys (61.8%) than girls (27.0%; missing 11.1%). Parents reported that 45.5% (n = 539) of the children have autism. Most respondents were a female primary caregiver (84.9%), and 78.0% were the biological mother of the cohort child with ID. The largest ethnic group for primary caregivers was White British (78.5%), over half were married and living with their partner (53.3%), and 39.3% were educated to degree level.

Findings to date: Data were collected on family, parental and child wellbeing, as well as demographic information. Wave 1 data collection took place between November 2015 and January 2017, primarily through online questionnaires. Telephone interviews were also completed by 644 primary caregivers.

Future plans: Wave 2 data collection is ongoing and the research team will continue following up these families in subsequent waves, subject to funding availability. Results will be used to inform policy and practice on family and child wellbeing in families of children with ID.

Keywords: Intellectual Disability, Family Research, Wellbeing, Longitudinal

For peer review only

ARTICLE SUMMARY

Strengths and limitations of this study

- A cohort of parental caregivers of children with ID to explore the wellbeing of children with ID, the wellbeing of parents and siblings, and the family system and relationships.
- The cohort includes over 1,000 families and includes children across the spectrum of ID severity.
- Data will be collected longitudinally over a number of years, allowing investigation of family experiences over time.
- For the first two study waves, data are based on parental report only.
- The cohort is likely to mainly include those who have the time and motivation to participate.

INTRODUCTION

Intellectual Disability (ID) is a condition described in ICD-11 as a Disorder of Intellectual Development[1]. Consistent with contemporary definitions of this condition, ID emerges during the "developmental period" (usually taken to mean before age 18 years), and is characterised by low cognitive ability (IQ <70) and low levels of adaptive behaviour (such as communication, social skills, independence skills - also assessed using standardised tools). Prevalence studies internationally suggest that approximately 3-4% of children and adolescents have an ID[2-3].

For several decades, researchers throughout the world have asked questions about whether the health (especially mental health) of family members of children with ID is different to that in families that are not raising a child with ID. Meta-analyses and results from higher quality research designs (such as population-based national samples) have suggested that mothers of children with ID are about 1.5 times more likely than other mothers to

For peer review only

experience depression[4], and similarly 1.4 times more likely than other mothers to have high levels of psychological symptoms indicative of mental health problems[5]. Data from the UK Millennium Cohort Study (a population representative birth cohort) also show that fathers of young children with ID are twice as likely to score above the cut-off on a psychiatric disorder screen when compared to fathers of other young children[6] and older siblings of young children with ID are 1.5 times more likely to score in the "abnormal" range on a screen for behavioural and emotional problems when compared to other siblings of young children[7].

Sometimes explicitly, but often implicitly, researchers making these comparisons between families of children with ID and those without are drawing on a family systems perspective that has been applied to families of children with a variety of severe cognitive and developmental disabilities[8-11]. The core idea is that families are systems and so changes in one part of the family system, or in one family sub-system (e.g., spousal, parent-child, sibling-sibling), will affect other family members and/or other family sub-systems.

Families of children with ID face multiple challenges. For example, children are more likely to, by definition, have cognitive and adaptive skill deficits, and are 4-5 times more likely to have mental health problems compared to other children[12]. Families are also at increased risk for multiple social/economic disadvantage including poverty and negative life events[12]. The day-to-day burden of care for a child with ID is high even when compared with other carers (e.g., dementia family carers)[13], but access to supports and services for children with ID and their families is limited or fraught with negative experiences often described as "battling" against the system[14,15].

Given these challenges facing families of children with ID, we might expect multiple negative effects on (for example) the psychological adjustment of parents and siblings. Research findings are generally consistent with this prediction. From a systems perspective, we would also expect family sub-systems to be negatively affected. Again, research evidence

For peer review only

supports this prediction. For example, using the population-based UK Millennium Cohort Study, we showed that parents of young children with ID reported more conflict and lower levels of closeness in the relationship with their child compared to other parents of young children[16]. In addition, meta-analytic reviews suggest that parents of children with disabilities, including ID, may be more likely to separate/divorce and have lower levels of satisfaction with their partner relationship compared to other parents[17,18]. We developed the 1,000 Families Study to explore these issues further and to extend current knowledge across several domains.

Study aims

The main aims of the 1,000 Families Study are:

1. To describe the wellbeing of families of children with ID and to compare these data wherever possible with normative data and/or to national datasets (especially the UK Millennium Cohort Study). Family members include mothers (or primary parental caregivers), fathers (or secondary parental caregivers), and siblings.
2. To examine wellbeing differences between mothers and fathers in families of children with ID.
3. To explore correlates of maternal, paternal and sibling wellbeing and relationship quality, including: deprivation and socio-economic circumstances; other child, parent, sibling and family demographic factors; other family members' wellbeing, and the child with ID's behaviour problems and adaptive skills.
4. To explore correlates of the behavioural and emotional wellbeing of the child with ID, including: family deprivation; other child, parent, sibling and family demographic factors; other family members' wellbeing, the child with ID's adaptive skills, and parenting attitudes and behaviours.

COHORT DESCRIPTION

For peer review only

To date, we have conducted an online cross-sectional survey and telephone interviews of over 1,000 families of children with ID who were living in the UK at the time of recruitment. Recruitment involved a multi-point contact method using websites, social media, advertisements in disability organisations' newsletters, and by approaching special schools and parent support organisations. To examine longitudinal associations, we are currently inviting these same families to participate in further data collection (Wave 2), which includes an online (or postal on request) survey but no telephone interview. To summarise the first and second waves of the 1,000 Families study:

Wave 1 data collection

The primary (and secondary if available) parental caregiver were asked to complete the same online survey: 1,184 primary caregivers and 93 secondary caregivers completed the Wave 1 survey between November 2015 and January 2017. The primary caregiver was also invited take part in a telephone interview following the questionnaire to provide information about the adaptive skills and behaviours of their child with ID. The telephone interview was arranged at a time convenient to the primary caregiver and most often took around an hour. The telephone interviews were completed by 644 primary caregivers. Differences between those who did and did not complete a telephone interview are discussed in Findings to Date.

Wave 2 data collection

Participants for Wave 2 are the caregivers who indicated during Wave 1 that they were willing to be contacted again to be invited to participate in Wave 2. The telephone interview was replaced with an additional measure on adaptive skills in the online survey. Caregivers are first contacted 2 years and 9 months after they completed Wave 1. Wave 2 data collection began in August 2018, and is anticipated to end in January 2021.

Study funding

For peer review only

The study is funded by Cerebra, a non-profit charity working with families with children with brain conditions. The project has also received funding from the Economic and Social Research Council Warwick Doctoral Training Centre, and the University of Warwick via PhD scholarship funding.

Study population

Inclusion criteria

1. For Wave 1, families with one or more children aged between 4 years and 15 years 11 months old who have an ID as reported by a parental caregiver. The child age range was initially (from November 2015) specified as 4 years to 11 years. In February 2017, an ethics amendment allowed us to expand recruitment to children aged up to 15 years and 11 months to meet families' interest in the study and to reach our sample target of over 1,000 families.
2. Families with at least one parental caregiver responding to the survey.
3. Families who live within the UK (i.e. England, Northern Ireland, Scotland or Wales).

Exclusion criteria

1. For Wave 1, families in which the child with ID lives outside of the family home on a full time basis (e.g., in a 52 week residential school placement).
2. Parental caregivers whose English literacy skills would not allow them to participate in an online survey and telephone interview.
3. Parental caregivers under 18 years old.

Wave 1 Cohort Description

Tables 1 and 2 summarise the key socio-demographic variables of the cohort child and their family respectively at Wave 1. All data were reported by the primary caregiver of the child with ID. These were usually the biological mother of the child with ID (78.0%) or another female relative (84.9% of the primary caregivers were female). Cohort children with

ID were aged between four years and 15 years and eleven months, with a mean age of 9.01 (SD = 2.93). There were more boys than girls (61.8% were male; 27.0% were female; 11.1% missing). The vast majority of families lived in England (*n* = 1,031), with fewer living in Scotland (*n* = 48), Wales (*n* = 83), and Northern Ireland (*n* = 9).

Table 1 provides some data indicative of ID severity in the cohort children. In terms of level of ID, 42.3% were reported by parents to have a severe to profound ID, and 42.2% a mild to moderate ID (15.5% missing). A mild to moderate ID was defined as "children with a mild to moderate intellectual disability can typically communicate and look after themselves well, but may take a bit longer to learn new skills compared to other children of the same age". A severe to profound ID was described as "children with a severe to profound intellectual disability are likely to have complex and multiple difficulties which require extensive support to learn and carry out daily activities". Inclusion policies in the UK schooling context means school-type is not precisely indicative of children's needs, particularly in primary schooling, nonetheless, 39.1% of children were attending a special school, in addition to 12.8% attending a mainstream school in a special provision or unit, and 28.1% attending a mainstream school. The Vineland Adaptive Behaviour Composite scores ranged from 25-100, with the mean score being 57.97 (*SD* = 11.13). The range of standard scores on the Vineland domains of Communication (25-117; mean = 62.02; *SD* = 13.63), Daily Living Skills (25-103; mean = 57.53; *SD* = 12.41), and Socialisation (15-110; mean = 59.24; *SD* = 12.40) were similarly wide. Thus, the full range of ID severity is represented within this cohort.

Common co-occurring conditions in the cohort of children with ID included autism (45.5%) and Down syndrome (13.6%). Other diagnoses were less common, but included Fragile X, Angelman syndrome, and numerous rare genetic conditions. A large number of children had additional physical health conditions or sensory impairments, including: visual

For peer review only

impairments (23.9%), hearing impairments (13.9%), epilepsy (12.5%), mobility problems (49.3%) and 'other physical health problems' (41%).

Measures

The study captured information on parental positivity, parental mental health, care burden/stress, life satisfaction, parent relationship, family satisfaction, parenting, child behavioural and emotional wellbeing, child adaptive behaviour, sibling behavioural and emotional wellbeing and sibling relationship quality. Tables 3 and 4 summarise the study measures, with number of missing items for each variable during Wave 1 indicated.

Patient and public involvement

The survey content and method was designed collaboratively with Cerebra, an organisation that supports and advocates for families of children with developmental disabilities, including ID. The final author (JM) participated in the design of the survey in her capacity as Cerebra staff member and PhD student at the University of Warwick. To date, the research team and Cerebra have collaborated to disseminate findings to families of children with ID through parent and practitioner research seminars that Cerebra regularly hosts.

Ethics

The study is being conducted in accordance with the ethical principles of the British Psychological Society. Approval was obtained from the National Health Service (NHS) West Midlands – South Birmingham Research Ethics Committee prior to commencement of the study. Sponsor approval was also obtained from the University of Warwick. There are no significant risks for the families from taking part in this study, but as some of the questions included in the survey ask about potentially challenging issues, such as parental and child wellbeing and difficulties, signposting to various resources and helplines has been provided in the information sheet and at the end of the survey for caregivers.

For peer review only

All participant information is stored in line with the requirements of the Data Protection Act 1998. While this study was approved and began before changes to the General Data Protection Regulation (GDPR) in 2018, data collection and procedures in Wave 2 are in line with GDPR requirements. Caregivers are informed through information sheets and

For peer review only

**Table 1.** Socio-economic and demographic information for Wave 1 Cerebra 1,000 Families sample: Child with ID	
**Child**	
Child age range (years)	4.01-15.92
Child age: mean (SD) (years)	9.01 (2.93)
Missing age n	175 (14.8%)
Child gender:	
n of males	732 (61.8%)
n of females	320 (27.0%)
Missing gender n	132 (11.1%)
Parental caregiver reported ID Level	
Severe to profound ID	501 (42.3%)
Mild to moderate ID	500 (42.2%)
Missing information	183 (15.5%)
Parental caregiver reported Autism diagnosis of child	539 (45.5%)
Down syndrome	161 (13.6%)
Cerebral palsy	87 (7.3%)
Child has a visual impairment	
Yes	283 (23.9%)
No	719 (60.7%)
Missing information	182 (15.4%)
Child has a hearing impairment	
Yes	164 (13.9%)
No	847 (71.5%)
Missing information	173 (14.6%)
Child has epilepsy	
Yes	148 (12.5%)
No	861 (72.7%)
Missing information	175 (14.8%)
Child has mobility problems	
Yes	584 (49.3%)
No	422 (35.6%)

For peer review only

Missing information	178 (15.0%)
Child has other physical health problems	
Yes	485 (41%)
No	523 (44.2%)
Missing information	176 (14.9%)
Vineland Adaptive Behaviour Composite (ABC)	
Mean (SD)	57.97 (11.14)
Range	25-100
Total responses	640 (54.1%)
Missing information	544 (45.9%)
Vineland Communication standard score	
Mean (SD)	62.02 (13.63)
Range	25-117
Total responses	642 (54.2%)
Missing information	542 (45.8%)
Vineland Daily Living Skills standard score	
Mean (SD)	57.53 (12.41)
Range	25-103
Total responses	643 (54.3%)
Missing information	541 (45.7%)
Vineland Socialisation standard score	
Mean (SD)	59.24 (12.40)
Range	15-110
Total responses	643 (54.3%)
Missing information	541 (45.7%)
Type of School:	
n of children attending special school	463 (39.1%)
n of children attending mainstream school	333 (28.1%)
n of children attending mainstream school in either a special unit or special educational needs provision	151 (12.8%)
Missing information	178 (15.0%)

For peer review only

**Table 2.** Socio-economic and demographic information for Wave 1 Cerebra 1,000 Families sample: Parent and Family	
**Primary Caregiver and Family**	
Female Primary Caregiver	1005 (84.9%)
Primary Caregiver Relationship to Child	
Biological Mother	923 (78%)
Adoptive Mother	53 (4.5%)
Biological Father	46 (3.9%)
Grandmother	13 (1.1%)
Missing information	129 (10.9%)
Primary Caregiver Employment status:	
In a job working for an employer	391 (33.0%)
Looking after home and family	388 (32.8%)
Self-employed	103 (8.7%)
Doing something else	100 (8.4%)
Missing information	129 (10.9%)
Primary caregiver education level:	
Degree level	465 (39.3%)
Higher education but below degree level	253 (21.4%)
A/AS Levels or equivalent	104 (8.8%)
5 or more GCSEs at A-C or equivalent*	74 (6.3%)
Some GCSEs passes or equivalent	93 (7.9%)
No qualifications	13 (1.1%)
Missing information	179 (15.1%)
Primary caregiver relationship status:	
Married and living with spouse/civil partner	631 (53.3%)
Divorced/separated/single/widowed/not currently living with partner	226 (19.1%)
Living with partner	141 (11.9%)
Missing information	186 (15.7%)

For peer review only

Primary Caregiver Ethnicity:	
White British	930 (78.5%)
White other (Irish, Travelling community, other)	55 (5.2%)
Asian/Asian British	26 (2.3%)
Black (African/Caribbean/Black British)	15 (1.3%)
Remaining ethnic groups (Mixed / multiple ethnicity, Arabic, Any other ethnic background etc.)	23 (2.0%)
Missing information	135 (11.4%)
Country:	
England	1031 (87.1%)
Scotland	48 (4.1%)
Wales	83 (7.0%)
Northern Ireland	9 (0.76%)
Missing	13 (1.10%)
Family living in most deprived 10% of neighbourhoods based on indices of multiple deprivation (IMD)	90 (7.6%)
Missing IMD information	34 (2.9%)
Child with ID has a sibling aged between 4 and 15 years 11 months	
Yes	612 (51.7%)
No	360 (30.4%)
Missing information	212 (17.9%)
Nearest-in-age sibling of child with ID has a longstanding illness, disability or infirmity	
Yes	168 (27.5%)
No	437 (71.4%)
Missing information (from those who indicated child has sibling in age range)	7 (1.1%)

For peer review only

Table 3. Parent and family measures used within the Cerebra 1,000 Families Study

Domain	Measure	Scoring	Measured at	Wave 1 Measure completeness
Parental positivity	Positive gains scale [Jess, Bailey, Pit-ten Cate, et al., Measurement invariance of the Positive Gains Scale in families of children with and without disabilities]<br>7 items<br>Perceived benefits for parent (5 items)<br>What family has gained (2 items)	5-point scale from 0 (*strongly agree*) to 4 (*strongly disagree*). Original scale had a lower score indicating higher positive gains. The scoring was reversed so that a higher score indicates higher positive gains.	W1, W2	Complete *n* = 1004<br>Missing *n* = 180
Parent mental health	Kessler 6 (K6)[19]<br>6 items	5-point Likert scale asks caregivers how often they've experienced six symptoms over the last 30 days from 0 (*none of the time*) to 4 (*all of the time*). Total score is calculated by summing the responses. A score of 13 and above indicates serious mental illness[20].	W1, W2	Complete *n* = 1000<br>Missing *n* = 184
Carer burden/stress	'Impact of care-giving on carer'<br>Survey of Informal Carers in Households 2009/2010[21]<br>7 items	Items ask individuals whether certain aspects of their lives have been affected by caring for another (*Yes* or *No*). Totals are generated for the responses, and higher scores indicate higher carer burden/stress.	W1, W2	Complete *n* = 963<br>Missing *n* = 221
Life satisfaction	Life satisfaction scale[22]<br>1 item	Single item measure that asks caregivers to rate their general life satisfaction from 1 (*completely dissatisfied*) to 10 (*completely satisfied*). Scores 6-10 indicate that respondents are 'satisfied overall'. Scores 4 and below indicate that participants are 'dissatisfied overall'.	W1, W2	Complete *n* = 998<br>Missing *n* = 186
Parent relationship – parent's perception of relationship with their partner (if applicable)	Disagreement over issues related to child[23]<br>1 item	This item asks caregivers to report on how often they disagree over issues relating to their child on a 6-point Likert scale (*Never* to *More than once a day*). There is also a response for 'Can't say'.	W1, W2	Complete *n* = 771<br>No live-in partner *n* = 226<br>Missing *n* = 187
	Happiness of relationship scale[23]<br>1 item	Caregivers are asked to rate how happy they are in their relationship from 1 (*very unhappy*) to 7 (*very happy*). There is also a response for 'Can't say'.	W1, W2	Complete *n* = 771<br>No live-in partner *n* = 226<br>Missing *n* = 187
Family functioning	Family APGAR scale[24]<br>5 items<br><br>Adaptability, Partnership, Growth, Affection and Resolve (APGAR)	Respondents are required to rate the frequency of feeling satisfied with each parameter on a 3-point Likert scale, from 0 (*hardly ever) to* 2 (*almost always*). The scale is scored by summing the 5 items. A higher score indicates a greater degree of satisfaction with family functioning (8-10 = *highly*	W1, W2	Complete *n* = 993<br>Missing *n* = 191

		functional, 4-7 = *moderately dysfunctional*, 0-3 = *dysfunctional*).		
Sibling behavioural and emotional wellbeing	Strengths and Difficulties Questionnaire (SDQ) (Parent questionnaire)[25]<br>25 items<br><br>Subscales: Emotional problems, Conduct problems, Hyperactivity/inattention, Peer relationship problems, Prosocial behaviour	Caregivers are asked to assess the extent to which each statement applies to the child based on the last 6 months using a 3-point rating scale (0 = *not true* to 2 = *certainly true*). The items are divided into 5 subscales. A total difficulties score can be obtained by summing the first four sub-scale scores: *close to average* (0-13), *slightly raised* (14-16), *high* (17-19), and *very high* (20-40).	W1, W2	Complete n = 603<br>No sibling n = 360<br>Missing n = 221
Sibling relationship	Sibling Relationship Questionnaire (SRQ) (Parent questionnaire) (revised)[26]<br>10 items<br>Adapted version using key items from two subscales of SRQ – SF:<br>Warmth and closeness (6 items)<br>Conflict (4 items)	Caregivers are asked about the sibling relationship on a 5-point Likert scale from 1 (*very much*) to 5 (*extremely much*). These items can then be used to derive measures that encapsulate positive or negative aspects of the sibling relationship.	W1, W2	SRQ warmth:<br>Complete n = 596<br>No sibling n = 360<br>Missing n = 228<br>SRQ conflict:<br>Complete n =599<br>No sibling n = 360<br>Missing n = 225
Parenting attitudes and behaviours	Child-parent relationship scale (CPRS)[27]<br>15 items<br>Conflict (8 items)<br>Closeness (7 items)	The caregiver states their feelings and beliefs about their relationship with their child and about the child's behaviour towards them, by responding to statements on a 5-point Likert scale (1 = *Definitely does not apply* to 5 = *Definitely applies*). CPRS generates a separate score for the individual conflicts and closeness constructs by summing the scores in each subscale	W1, W2	Closeness:<br>Complete n = 978<br>Missing n = 206<br>Conflict:<br>Complete n = 983<br>Missing n = 201
	Alabama Parenting questionnaire (Parent form)[28]<br>12 items<br>Positive parenting (6 items) Inconsistent discipline (6 items)	Parents/caregivers are asked about their parenting behaviours on a five-point Likert scale (1 = *Never* to 5 = *Always*). The items in each scale are summed to obtain two scale scores.	W1, W2	Positive parenting:<br>Complete n = 972<br>Missing n = 212<br>Inconsistent discipline:<br>Complete n = 961<br>Missing n = 223
	Parent-child activities index<br>5 items	This questionnaire was constructed for the purposes of this study. Some of the questions have been used in national UK surveys such as the Millennium Cohort Study, and where relevant, items were adapted to be more appropriate for families of children with ID. The questions ask about parent-child shared activities. Answers are provided on a 5-point Likert scale (from 1 = *Not at all* to 5 = *Every day*).	W1<br>*Removed from W2 due to parental feedback.*	Complete n = 974<br>Missing n = 210

Note. W1 and W2 indicates Wave 1 and Wave 2

For peer review only - http://bmjopen.bmj.com/site/about/guidelines.xhtml

**Table 4.** Child measures used within the Cerebra 1,000 Families Study				
**Domain**	**Measure**	**Scoring**	**Measured at**	**Wave 1 Measure completeness**
Child with ID behavioural and emotional wellbeing	Strengths and Difficulties Questionnaire (SDQ) (Parent questionnaire)[25]<br>25 items<br>Subscales: Emotional problems, Conduct problems, Hyperactivity/inattention, Peer relationship problems, Prosocial behaviour	Caregivers are asked to assess the extent to which each statement applies to the child based on the last 6 months using a 3-point rating scale (0 = *not true* to 2 = *certainly true*). The items are divided into 5 subscales. A total difficulties score can be obtained by adding the first four sub-scale scores and categorising them into a four band system: *close to average* (0-13), *slightly raised* (14-16), *high* (17-19), and *very high* (20-40).	W1, W2	Complete *n* = 985<br>Missing *n* = 199
Behavioural and emotional problems of the child with ID	Development behaviour checklist (parent version) (DBC-P)[29]<br>96 items<br>Telephone interview<br>Subscales: Disruptive/Anti-social Behaviour, Self-absorbed, Communication Disturbance, Anxiety, Social Relating	Items are scored on a 0, 1 or 2 rating scale (0 = *Not true as far as you know*, 1 = *Somewhat or sometimes true*, 2 = *Very true or often true*). The Total Behaviour Problem Score (TBPS) is an overall measure of emotional and behavioural problems, and can detect clinically significant levels of overall emotional and behavioural disturbance (indicated by a score of 46 or greater).	W1	Complete *n* = 644<br>Did not consent to telephone interview *n* = 198<br>Missing *n* = 342
Adaptive behaviour skills of the child with ID	Vineland adaptive behaviour scales – second edition (parent interview form) (VABS- II)[30]<br>30-40 minute telephone interview<br><br>Communication Skills<br>Daily Living Skills<br>Socialisation Skills	An overall composite score, and domain scores, can be derived with reference to age during typical development during which children can perform the task items. Four standardised scores in total were derived for the present research: Adaptive Behaviour Composite, Communication Skills, Daily Living Skills, and Socialisation Skills. Age Equivalency scores were also calculated.	W1	Complete *n* = 645<br>Did not consent to telephone interview *n* = 198<br>Missing *n* = 341
Adaptive behaviour skills of the child with ID	The GO4KIDDS Brief Adaptive Scale[31]<br>9 items	Items cover communication, self-help skills, social interaction, and support needs. Each item is rated on a 5-point scale where higher scores indicate greater skill level and greater independence. These item scores are summed to get an overall Adaptive Behaviour score. We have amended the original GO4KIDDS to include an additional item asking about alternative methods of communication following feedback from caregivers.	W2<br><br>Replaced the VABS-II interview	Not yet known

Note. W1 and W2 indicates Wave 1 and Wave 2

consent forms on what data are collected, why this is necessary, and how these data are stored. All aspects of the study are conducted on the basis of explicit written consent to take part in each stage of the study. All research data are stored electronically and password protected on firewalled University computers with access limited to the research team. The password and access to the research data, including participant information, is restricted to the Chief Investigator and the research team.

For Wave 2 of the data collection, consent is collected as it was in Wave 1 and participants are asked again if they would be willing to be contacted for any future research. At Wave 2, a £10 gift voucher was introduced as a Thank You to caregivers who complete the survey. To mitigate against potential biases arising from the monetary incentive, it is made clear in the information sheet that caregivers do not have to answer any questions that make them feel uncomfortable and they will still receive the voucher.

Dissemination

Throughout the study, results will be published in peer-reviewed academic journals. Presentations of study findings will be made at research conferences and seminars. The charity Cerebra and other stakeholders will be involved in methods of dissemination for reaching the wider community, particularly families of children with ID. Caregivers who took part in the study, and have consented, receive updates about the study through a newsletter and social media, which the public can also access. This allows findings to be summarised and presented in an accessible format in addition to any academic papers or presentations.

FINDINGS TO DATE

No findings have yet been reported from the data on this cohort. Overall, the parent-reported severity of ID, educational setting, and VABS scores indicate that this cohort includes children across a wide range of ID severity, although the VABS scores indicate more children

For peer review only

in the mild-moderate range, but this would be expected given a lower prevalence for severe to profound ID.

Telephone interviews were completed by 644 of the primary caregivers indicating that families who completed both the survey and the telephone interview may have had access to more resources that enable them to contribute more time to research participation. For instance, deprivation levels may be associated with telephone interview participation. To identify family deprivation, we constructed a composite variable with values ranging from zero to four, incorporating measures of: subjective poverty, hardship, income poverty, and neighbourhood deprivation. Primary caregivers that did not complete telephone interviews had a mean deprivation composite score of 1.71 (*SD* = 1.10) compared to a mean deprivation composite score of 1.37 (SD = 1.09) for primary caregivers that completed a telephone interview ($t(948) = 4.72$; $p < .001$; $d = .32$; MD = 0.35, 95% CI .20, .49). Primary caregivers who completed the telephone interviews were more likely to have a higher education qualification (75.1%) compared to primary caregivers that did not complete a telephone interview (66.6%; $\chi 2(1, N = 1{,}002) = 8.58$; $p = .003$; $\phi = .093$). Primary caregivers who were single parents provided a telephone interview in 53.5% of cases, compared to primary caregivers from two-parent households, who provided a telephone interview in 65.3% of cases ($\chi 2(1, N = 998) = 10.30$; $p = .001$; $\phi = -.102$.

In addition to resources, differences between those who participated in telephone interviews and those who did not included differences in child profiles. Children of caregivers who did not take part in the telephone interview had higher total SDQ total behaviour scores with a mean score of 22.00 (*SD* = 6.52) compared to a mean score of 20.63 (*SD* = 6.48) for those who did participate ($t(983) = 3.201$; p .001; $d = .21$; $MD = 1.38$, 95% CI .53, 2.22). Primary caregivers who did not complete the telephone interviews had children with ID that had fewer coexisting physical health problems (such as epilepsy, mobility problems, hearing

For peer review only

or visual problems; mean = 1.49; *SD* = 1.22) compared to the children with ID of primary caregivers that did complete the telephone interviews (mean = 1.76; *SD* = 1.33; $t(985) = -3.157$; p .002; $d = .21$; *MD* = -0.27, 95% CI -.43, -.10).

Comparison with indicators from the 2011 census of England and Wales[32] indicate lower participation of families identifying as Black, Asian or Minority Ethnic (5.6% of the 1,000 Families sample compared to 13% of the population of England and Wales), but expected numbers of families identifying as White British (78.5% of the 1,000 Families sample, compared to 80.5% in the 2011 Census) (see Table 2 for full ethnicity information on the 1,000 Families). In terms of educational qualifications, the survey sample were more highly educated (39.1% of the primary caregivers in the 1,000 Families were educated to degree level, in comparison to 27% in the England and Wales 2011 Census)[32]. Whilst 23% of the population in England and Wales had no educational qualifications, only 1.1% of the 1,000 Families sample reported having no educational qualifications. These comparisons to the census data should be viewed with caution as the census data about qualifications include the full age-range of adults living in England and Wales in 2011, whereas our sample of primary caregivers disproportionately includes women of childrearing age across all four countries of the UK. Policy changes leading to the expansion of UK higher education in recent decades means we would expect that the primary caregivers in our sample were more likely to have completed degrees than were previous generations. Furthermore, the 2011 census data included people aged 16-18 and this group were less likely to have any qualifications yet, although they may have been in education. In terms of socio-economic indicators, 7.6% of the families in the 1,000 Families sample were living in the most deprived areas in their respective country based on the indices of multiple deprivation for England, Scotland, Wales and Northern Ireland [33-36]. These indices offer a measure of relative deprivation at a local scale by combining information on income poverty, education,

For peer review only

unemployment, health, crime, barriers and access to services, housing, and physical environment for each small area and then rank-ordering areas within each country.

Future directions

The data will be analysed in cross-sectional and longitudinal studies. Where possible, data will be compared with data from normative or population-representative samples. Correlation and regression approaches will be used to explore factors associated with the wellbeing of mothers, fathers, siblings, and the child with ID. Where appropriate, models will account for the nested nature of data within families. The cohort provides scope to explore a range of research questions, for example, studies exploring: associations between behavioural outcomes for children with ID and their siblings in relation to their sibling relationship quality, anxiety in children with ID with and without autism, and wellbeing in mothers of children with Down syndrome. The cross-sectional studies using data from Wave 1 will also help to develop models and questions that can be explored through longitudinal research following future waves.

STRENGTHS AND LIMITATIONS

This study is, as far as we are aware, the largest cohort of families with a child with ID in the UK and will make an important contribution to the research on the wellbeing of children with ID and their families. Particularly important is the longitudinal design which currently includes a second wave of data collection that is underway, with a third wave under development. This adds to the growing global research following families of children with ID and/or autism over time[37-38]. Selection of measures allows for some comparison with population-representative samples in the UK (e.g. the Millennium Cohort Study[39]). Results will inform parents, clinicians, and educational professionals on factors related to the wellbeing of children with ID, their parents, and their siblings.

For peer review only

Designing this research study via a questionnaire and telephone interview allowed us to recruit a large sample from across the UK. However, this precluded an independent ascertainment of child ID, and/or associated developmental conditions (e.g., autism). Instead, information on the child is based solely on parental report, although this includes their educational setting, parent-reported severity of ID, any diagnoses, and for those who took part in the telephone interview, a measure of child adaptive behaviour. The implication of this limitation is that the survey cannot verify the extent of clinical diagnoses available for each child with ID. Where research evidence has examined the validity of parental reports, it has suggested that parents' reports correspond well to clinical diagnoses available[40, 41] and that parental reports on children's developmental outcomes are accurate[42].

Although the sample size recruited at Wave 1 is large (>1,000 families), the sampling design (convenience sample recruited through social media and ID-related organisations) may have resulted in sampling bias, where families of children with ID in contact with charitable organisations and/or access to social media may be more likely to be included. However, although not fully representative of the UK, the findings discussed previously indicate that the 1,000 Families sample includes elements of diversity that have the potential to provide insights into families with a child with ID from various backgrounds.

Another limitation of this study is that not all primary caregivers completed a telephone interview as well as the survey – 644 out of 1,184 primary caregivers completed a telephone interview. As previously summarised in the Findings to Date section, there were demographic differences between these groups. For example, those primary caregivers that did not take part in the telephone interview had higher levels of poverty, lower levels of qualifications, were more likely to be a single parent, and reported higher average SDQ total behaviour scores for their children with ID. The children of the primary caregivers that did

For peer review only

not complete the telephone interviews had fewer co-existing physical health problem labels compared to the children of primary caregivers that did complete the telephone interviews.

Last, although 772 of the primary caregivers in the sample indicated they were married and/or living with a partner, only 93 secondary caregivers participated in the survey. Overall low rates of participation of fathers or secondary caregivers in family research is a well-described phenomenon that may relate to role allocation between parents regarding child care, working commitments, and primary responsibility for contact with disability and educational services[43-47]. The 1,000 Families study directly recruited and encouraged fathers to take part in the study, but it is likely that a recruitment strategy primarily focused on the family was not specific enough to encourage greater participation from fathers and secondary caregivers. There is a need for further investigation on why other family members, particularly fathers, were not as likely to take part, and whether there are recruitment strategies which are more successful to reach fathers and secondary caregivers, as some research has highlighted the importance of inviting fathers directly and emphasizing the importance of their participation[47, 43].

COLLABORATION

Initial data analyses and publications will be generated by researchers at CEDAR, University of Warwick as part of the 1,000 Families study funded by Cerebra. However, the research team welcome researchers interested in future collaboration to contact the corresponding author with their expression of interest. Access and analysis of the data is currently only possible via the University of Warwick, due to the ethical approvals in place.

Acknowledgements We would like to thank all the families that participated in this study. We acknowledge the support from Cerebra in funding, developing and recruiting to this

For peer review only

study; and the ESRC Doctoral Training Centre at the University of Warwick for funding support in the form of PhD studentships. We acknowledge the contributions of our study administrators, Alison Baker and Diana Smith. Various researchers assisted in conducting telephone interviews, alongside the authors, to collect data for Wave 1 of this study and we acknowledge their support: Latoya Clarke, Farah Elahi, Gemma Gray, Liz Halstead, Sasha Mandair, Louise Rixon, Suzi Scott and Ruth Thomas.

Author Contributions RH and VT conceptualised and co-designed the study, wrote the study protocol, contributed to the drafting of the manuscript for publication, reviewed and revised the manuscript, and read and approved the final manuscript. NH and CM contributed to the design of the study, contributed to the drafting of the manuscript for publication, reviewed and revised the manuscript, and read and approved the final manuscript. MJ, EL and JM contributed to the design of the study, contributed to the original protocol, and read and approved the final manuscript.

Funding statement The authors acknowledge the funding support of Cerebra, UK, the Economic and Social Research Council Warwick Doctoral Training Centre, and the University of Warwick.

Competing Interests Statement None declared.

Patient consent Parental/guardian consent obtained.

Data Availability Statement Data from this study are not available for sharing due to ethical approval requirements. Researchers interested in collaboration should contact the corresponding author with their expression of interest.

Ethics approval: Full ethical approval was granted for this study from the UK National Health Service (NHS) West Midlands – South Birmingham Research Ethics Committee: REC reference number: 15/WM/0267 (11th September 2015). An amendment was granted to extend the age range (7th March 2017). An amendment was granted for changes made to

For peer review only

Wave 2 (8th February 2018) and the ethics committee was informed of further minor changes made to the documents for Wave 2 (27th February 2018). Sponsor approval was also obtained from the University of Warwick.

For peer review only

REFERENCES

1. Salvador-Carulla L, Reed GM, Vaez-Azizi, LM, *et al.* Intellectual developmental disorders: towards a new name, definition and framework for "mental retardation/intellectual disability" in ICD-11. *World Psychiatry* 2011;10(3):175-180. doi:10.1002/j.2051-5545.2011.tb00045.x2Platt JM, Keyes KM, McLaughlin KA, Kaufman AS. Intellectual disability and mental disorders in a US population representative sample of adolescents. *Psychol Med*. 2019;49(6):952–961. doi:10.1017/S0033291718001605

3. Anderson LL, Larson SA, MapelLentz S, Hall-Lande J. A Systematic Review of U.S. Studies on the Prevalence of Intellectual or Developmental Disabilities Since 2000. *Intellect Dev Disabil.* 2019;57(5):421-438. doi: 10.1352/1934-9556-57.5.421.

4. Singer GH. Meta-analysis of comparative studies of depression in mothers of children with and without developmental disabilities. *Am J Ment Retard* 2006;111:155-169. doi:10.1352/0895-8017(2006)111[155:MOCSOD]2.0.CO;2

5. Emerson E. Mothers of children and adolescents with intellectual disability: Social and economic situation, mental health status, and the self-assessed social and psychological impact of the child's difficulties. *J Intellect Disabil Res* 2003;47:385-399. doi:10.1046/j.1365-2788.2003.00498.x

6. Emerson E, McCulloch A, Graham H, *et al.* Socioeconomic circumstances and risk of psychiatric disorders among parents of children with early cognitive delay. *Am J Intellect Dev Disabil* 2010;115:30-42. doi:10.1352/1944-7558-115.1.30

7. Hayden NK, Hastings RP, Totsika V, *et al.* A Population-Based Study of the Behavioral and Emotional Adjustment of Older Siblings of Children with and without Intellectual Disability. *J Abnorm Child Psychol* Published Online First: 4 February 2019. doi:10.1007/s10802-018-00510-5

For peer review only

8. Cridland EK, Jones SC, Magee CA, *et al.* Family-focused autism spectrum disorder research: a review of the utility of family systems approaches. *Autism* 2014;18:213-222. doi:10.1177/1362361312472261

9. Kovshoff H, Cebula K, Tsai H-WJ, *et al.* Siblings of children with autism: The Siblings Embedded Systems Framework. *Curr Dev Disord Rep* 2017;4(2):37-45. doi:10.1007/s40474-017-0110-5

10. Seligman M, Darling RB. *Ordinary families, special children.* 3rd ed. New York: Guilford Press, 2007.

11. Trivette CM, Dunst CJ, Hamby DW. Influences of family-systems intervention practices on parent-child interactions and child development. *Topics Early Child Spec Educ* 2010;30:3-19. doi:10.1177/0271121410364250

12. Emerson E, Hatton C. Mental health of children and adolescents with intellectual disabilities in Britain. *Br J Psychiatry* 2007;191:493–499. doi:10.1192/bjp.bp.107.038729

13. Totsika V, Hastings RP, Vagenas D. Informal caregivers of people with an intellectual disability in England: Health, quality of life and impact of caring. *Health Soc Care Community* 2017:25:951-961. doi:10.1111/hsc.12393

14. Griffith GM, Hastings RP. “He’s hard work, but he’s worth it.” The experience of caregivers of individuals with intellectual disabilities and challenging behaviour: A meta-synthesis of qualitative research. *J Appl Res Intellect Disabil* 2014;27:401-419. doi:10.1111/jar.12073

15. Toms G, Totsika V, Hastings RP, *et al.* Access to services by children with intellectual disability and mental health problems: Population-based evidence from the UK. *J Intellect Dev Disabil* 2015;40:239-247. doi:10.3109/13668250.2015.1045460

16. Totsika V, Hastings RP, Vagenas D, *et al.* Parenting and the behavior problems of young children with an intellectual disability: Concurrent and longitudinal relationships in a

For peer review only

population-based study. *Am J Intellect Dev Disabil* 2014;119:422-435. doi:10.1352/1944-7558-119.5.422

17. Risdal D, Singer GHS. Marital Adjustment in Parents of Children with Disabilities: A Historical Review and Meta-Analysis. *Res Pract Persons Severe Disabl* 2004;29:95-103. doi:10.2511/rpsd.29.2.95

18. Saini M, Stoddart KP, Gibson M, *et al.* Couple relationships among parents of children and adolescents with Autism Spectrum Disorder: Findings from a scoping review of the literature. *Res Autism Spectr Disord* 2015;17:142-157. doi:10.1016/j.rasd.2015.06.014

19. Kessler RC, Andrews G, Colpe LJ, *et al.* Short screening scales to monitor population prevalences and trends in non-specific psychological distress. *Psychol Med* 2002;32:959-976. doi:10.1017/S0033291702006074

20. Kessler RC, Barker PR, Colpe LJ, *et al.* Screening for serious mental health difficulties in the general population. *Arch Gen Psychiatry* 2003;60:184-189 doi:10.1001/archpsyc.60.2.184

21. The NHS Information Centre, Social Care team. 451845 Survey of Informal Carers in Households 2009/10 Mainstage questionnaire [Internet]. NHS Information Centre; Leeds, 2010. p. 24. Available from: https://files.digital.nhs.uk/publicationimport/pub02xxx/pub02200/surv-care-house-care-ques-eng-2009-2010-apx2.pdf

22. Department for Environment, Food and Rural Affairs (DEFRA). Life satisfaction and other measures of wellbeing in England, 2007-2011 [Internet]. DEFRA; London, 2011. Available from: https://webarchive.nationalarchives.gov.uk/20130123225714/http://www.defra.gov.uk/statistics/files/Statistical-Release-13-April-2011-wellbeing.pdf

For peer review only

23. University of London, Institute of Education, Centre for Longitudinal Studies. Millennium Cohort Study Second Survey, 2003-2005: CAPI Questionnaire Documentation. [Internet]. Centre for Longitudinal Studies; London, 2007. p. 96-97. Available from: http://doc.ukdataservice.ac.uk/doc/5350/mrdoc/pdf/mcs2_capi_questionnaire_documentation_november_2007.pdf

24. Smilkstein G. The Family APGAR: A proposal for a family function test and its use by physicians. *The Journal of Family Practice* 1978;6:1231-1239.

25. Goodman R. The Strengths and Difficulties Questionnaire: A research note. *J Child Psychol Psychiatry* 1997;38:581-586. doi:10.1111/j.1469-7610.1997.tb01545.x

26. Furman W, Buhrmester D. Children's perceptions of the qualities of sibling relationships. *Child Development* 1998;56:448-461. doi:10.2307/1129733

27. Pianta RC. *Child-Parent Relationship Scale*. Charlottesville, VA: University of Virginia, 1995.

28. Frick PJ. The Alabama Parenting Questionnaire. Unpublished rating scale, University of Alabama, 1991.

29. Einfeld SL, Tonge BJ. *Manual for the Developmental Behaviour Checklist: Primary Carer Version (DBC-P) & Teacher Version (DBC-T) (2nd ed.)*. Clayton, Melbourne: Monash University Centre for Developmental Psychiatry and Psychology, 2002.

30. Sparrow SS, Cicchetti DV, Saulnier CA. *Vineland Adaptive Behavior Scales, Survey Forms Manual*. 3rd ed. Circle Pines, MN: AGS Publishing, 2016.

31. Perry A, Taheri A, Ting V, *et al.* The GO4KIDDS Brief Adaptive Scale. *J Appl Res Intellect Disabil* 2015;28:594-597. doi:10.1111/jar.12143

32. Ethnicity in the UK. *GOV.UK website, Ethnicity facts and figures* 2019. https://www.ethnicity -facts-figures.service.gov.uk/ethnicity-in-the-uk. Accessed 9th March 2019.

For peer review only

33. Gill B. *The English Indices of Deprivation 2015: Statistical Release*. London: Department for Communities and Local Government, 2015. https://www.gov.uk/government /statistics/english-indices-of-deprivation-2015. Accessed 16th June 2019.

34. *Introducing the Scottish Index of Multiple Deprivation 2016.* Edinburgh: APS Group Scotland for The Scottish Government, 2016. https://www2.gov.scot.Resource/ 0050/00504809.pdf Accessed 16th June 2019.

35. *Welsh Index of Multiple Deprivation 2014: Executive summary revised.* Knowledge and Analytical Services, Welsh Government, 2015. https://gweddill.gov.wales/docs/statistics /2015/150812-wimd-2014-summary-revised-en.pdf. Accessed 16th June 2019.

36. *Northern Ireland Multiple Deprivation Measures 2017: Technical Report.* Northern Ireland Statistics and Research Agency, 2017. https://www.nisra.gov.uk/sites/ nisra.gov.uk/files/publications/NIMDM%202017_Technical%20Report.pdf. Accessed 16th June 2019.

37. Woodman AC, Smith LE, Greenberg JS, *et al.* Change in autism symptoms and maladaptive behaviors in adolescence and adulthood: the role of positive family processes. *J Autism Dev Disord* 2015;45(1):111-26. doi: 10.1007/s10803-014-2199-2.

38. Smith LE, Greenberg JS, Mailick MR. The family context of autism spectrum disorders: influence on the behavioral phenotype and quality of life. *Child Adolesc Psychiatr Clin N Am*. 2014;23(1):143–155. doi:10.1016/j.chc.2013.08.006

39. University of London, Institute of Education, Centre for Longitudinal Studies.. Millennium Cohort Study: Fourth Survey, 2008. [data collection]. 7th Edition. UK Data Service, 2017. SN: 6411. doi:10.5255/UKDA-SN-6411-7

40. Daniels AM, Rosenberg RE, Anderson C, *et al.* Verification of parent-report of child autism spectrum disorder diagnosis to a web-based autism registry. *J Autism Dev Disord* 2012;42(2):257-265. doi:10.1007/s10803-011-1236-7

For peer review only

41. Lee H, Marvin AR, Watson T, *et al*. Accuracy of Phenotyping of Autistic Children Based on Internet Implemented Parent Report. *Am J Med Genet **B** Neuropsychiatr Genet* 2010;0(6):1119–1126. doi:10.1002/ajmg.b.31103

42. Charman T, Howlin P, Berry B, *et al*. Measuring developmental progress of children with autism spectrum disorder on school entry using parent report. *Autism* 2004;8(1):89-100. doi:10.1177/1362361304040641

43. Costigan, CL, Cox, MJ. Fathers' participation in family research: is there a self-selection bias? *J Fam Psychol* 2001;15(4):706-720.

44. Cassano M, Adrian M, Veits G, *et al.* The inclusion of fathers in the empirical investigation of child psychopathology: an update. *J Clin Child Adolesc Psychol* 2006;35(4):583-589. doi:10.1207/s15374424jccp3504_10

45. Mitchell SJ, See HM, Tarkow AKH, *et al.* Conducting studies with fathers: Challenges and opportunities. *Appl Dev Sci* 2007;11(4):239–244. doi:10.1080/10888690701762159

46. Doyle O, Weller BE, Daniel SS, *et al.* Overcoming Barriers to Fathers' Participation in Clinically Relevant Research: Recommendations from the Field, *Social Work Research* 2016;40(4):260–264. doi:10.1093/swr/svw015

47. Phares V, Lopez E, Fields S, *et al*. Are fathers involved in pediatric psychology research and treatment? *J Pediatr Psychol* 2005;30(8):631-643. DOI: 10.1093/jpepsy/jsi050

For peer review only

BMJ Open

Cohort Profile: The 1,000 Families Study, a UK multi-wave cohort investigating the wellbeing of families of children with intellectual disabilities.

Journal:	*BMJ Open*
Manuscript ID	bmjopen-2019-032919.R2
Article Type:	Cohort profile
Date Submitted by the Author:	08-Jan-2020
Complete List of Authors:	Hastings, Richard; University of Warwick, Centre for Educational Development, Appraisal and Research (CEDAR); Monash University, Centre for Developmental Psychiatry and Psychology, Department of Psychiatry<br>Totsika, Vasiliki; University College London, Division of Psychiatry; University of Warwick, Centre for Educational Development, Appraisal and Research (CEDAR)<br>Hayden, Nikita; University of Warwick, Centre for Educational Development, Appraisal and Research (CEDAR)<br>Murray, Caitlin; University of Warwick, Centre for Educational Development, Appraisal and Research (CEDAR)<br>Jess, Mikeda; University of Warwick, Centre for Educational Development, Appraisal and Research (CEDAR)<br>Langley, Emma; University of Warwick, Centre for Educational Development, Appraisal and Research (CEDAR)<br>Margetson, Jane; University of Warwick, Centre for Educational Development, Appraisal and Research (CEDAR); Cerebra
**Primary Subject Heading**:	Mental health
Secondary Subject Heading:	Public health, Paediatrics
Keywords:	Family Research, Intellectual Disability, Wellbeing, Longitudinal

SCHOLARONE™
Manuscripts

I, the Submitting Author has the right to grant and does grant on behalf of all authors of the Work (as defined in the below author licence), an exclusive licence and/or a non-exclusive licence for contributions from authors who are: i) UK Crown employees; ii) where BMJ has agreed a CC-BY licence shall apply, and/or iii) in accordance with the terms applicable for US Federal Government officers or employees acting as part of their official duties; on a worldwide, perpetual, irrevocable, royalty-free basis to BMJ Publishing Group Ltd ("BMJ") its licensees and where the relevant Journal is co-owned by BMJ to the co-owners of the Journal, to publish the Work in this journal and any other BMJ products and to exploit all rights, as set out in our licence.

The Submitting Author accepts and understands that any supply made under these terms is made by BMJ to the Submitting Author unless you are acting as an employee on behalf of your employer or a postgraduate student of an affiliated institution which is paying any applicable article publishing charge ("APC") for Open Access articles. Where the Submitting Author wishes to make the Work available on an Open Access basis (and intends to pay the relevant APC), the terms of reuse of such Open Access shall be governed by a Creative Commons licence – details of these licences and which Creative Commons licence will apply to this Work are set out in our licence referred to above.

Other than as permitted in any relevant BMJ Author's Self Archiving Policies, I confirm this Work has not been accepted for publication elsewhere, is not being considered for publication elsewhere and does not duplicate material already published. I confirm all authors consent to publication of this Work and authorise the granting of this licence.

For peer review only

Cohort Profile: The 1,000 Families Study, a UK multi-wave cohort investigating the wellbeing of families of children with intellectual disabilities

Author names

Richard P. Hastings[1, 2], Vasiliki Totsika[3, 2, 1], Nikita K. Hayden[1], Caitlin A. Murray[1], Mikeda Jess[1], Emma Langley[1], & Jane Margetson[1, 4].

Email addresses: R.Hastings@warwick.ac.uk, V.Totsika@ucl.ac.uk, N.Hayden@warwick.ac.uk, C.Murray.7@warwick.ac.uk, M.Jess@warwick.ac.uk, E.Langley@warwick.ac.uk, J.Margetson@warwick.ac.uk

Corresponding author: Caitlin A. Murray (C.Murray.7@warwick.ac.uk, 024 76575866)

Author affiliations

[1] Centre for Educational Development, Appraisal and Research, University of Warwick, Coventry, UK

[2] Centre for Developmental Psychiatry and Psychology, Department of Psychiatry, Monash University, Melbourne, Australia

[3] Division of Psychiatry, University College London, London, UK

[4] Cerebra, Carmarthen, UK

Word count: 4073

For peer review only

ABSTRACT

Purpose: The 1,000 Families study is a large, UK-based, cohort of families of children with intellectual disabilities (ID). The main use of the cohort data will be to describe and explore correlates of the wellbeing of families of children with ID, including parents and siblings, using cross-sectional and (eventually) longitudinal analyses. The present cohort profile intends to describe the achieved cohort.

Participants: Over 1,000 families of UK children with ID aged between 4 years and 15 years 11 months (total N = 1,184) have been recruited. The mean age of the cohort was 9.01 years old. The cohort includes more boys (61.8%) than girls (27.0%; missing 11.1%). Parents reported that 45.5% (*n* = 539) of the children have autism. Most respondents were a female primary caregiver (84.9%), and 78.0% were the biological mother of the cohort child with ID. The largest ethnic group for primary caregivers was White British (78.5%), over half were married and living with their partner (53.3%), and 39.3% were educated to degree level.

Findings to date: Data were collected on family, parental and child wellbeing, as well as demographic information. Wave 1 data collection took place between November 2015 and January 2017, primarily through online questionnaires. Telephone interviews were also completed by 644 primary caregivers.

Future plans: Wave 2 data collection is ongoing and the research team will continue following up these families in subsequent waves, subject to funding availability. Results will be used to inform policy and practice on family and child wellbeing in families of children with ID. As this cohort profile aims to describe the cohort, future publications will explore relevant research questions and report key findings related to family wellbeing.

Keywords: Intellectual Disability, Family Research, Wellbeing, Longitudinal

For peer review only

ARTICLE SUMMARY

Strengths and limitations of this study

- A cohort of parental caregivers of children with ID to explore the wellbeing of children with ID, the wellbeing of parents and siblings, and the family system and relationships.
- The cohort includes over 1,000 families and includes children across the spectrum of ID severity.
- Data will be collected longitudinally over a number of years, allowing investigation of family experiences over time.
- For the first two study waves, data are based on parental report only.
- The cohort is likely to mainly include those who have the time and motivation to participate.

INTRODUCTION

Intellectual Disability (ID) is a condition described in ICD-11 as a Disorder of Intellectual Development[1]. Consistent with contemporary definitions of this condition, ID emerges during the “developmental period” (usually taken to mean before age 18 years), and is characterised by low cognitive ability (IQ <70) and low levels of adaptive behaviour (such as communication, social skills, independence skills - also assessed using standardised tools). Prevalence studies internationally suggest that approximately 3-4% of children and adolescents have an ID[2-3].

For several decades, researchers throughout the world have asked questions about whether the health (especially mental health) of family members of children with ID is different to that in families that are not raising a child with ID. Meta-analyses and results from higher quality research designs (such as population-based national samples) have suggested that mothers of children with ID are about 1.5 times more likely than other mothers to

For peer review only

experience depression[4], and similarly 1.4 times more likely than other mothers to have high levels of psychological symptoms indicative of mental health problems[5]. Data from the UK Millennium Cohort Study (a population representative birth cohort) also show that fathers of young children with ID are twice as likely to score above the cut-off on a psychiatric disorder screen when compared to fathers of other young children[6] and older siblings of young children with ID are 1.5 times more likely to score in the "abnormal" range on a screen for behavioural and emotional problems when compared to other siblings of young children[7].

Sometimes explicitly, but often implicitly, researchers making these comparisons between families of children with ID and those without are drawing on a family systems perspective that has been applied to families of children with a variety of severe cognitive and developmental disabilities[8-11]. The core idea is that families are systems and so changes in one part of the family system, or in one family sub-system (e.g., spousal, parent-child, sibling-sibling), will affect other family members and/or other family sub-systems.

Families of children with ID face multiple challenges. For example, children are more likely to, by definition, have cognitive and adaptive skill deficits, and are 4-5 times more likely to have mental health problems compared to other children[12]. Families are also at increased risk for multiple social/economic disadvantage including poverty and negative life events[12]. The day-to-day burden of care for a child with ID is high even when compared with other carers (e.g., dementia family carers)[13], but access to supports and services for children with ID and their families is limited or fraught with negative experiences often described as "battling" against the system[14,15].

Given these challenges facing families of children with ID, we might expect multiple negative effects on (for example) the psychological adjustment of parents and siblings. Research findings are generally consistent with this prediction. From a systems perspective, we would also expect family sub-systems to be negatively affected. Again, research evidence

For peer review only

supports this prediction. For example, using the population-based UK Millennium Cohort Study, we showed that parents of young children with ID reported more conflict and lower levels of closeness in the relationship with their child compared to other parents of young children[16]. In addition, meta-analytic reviews suggest that parents of children with disabilities, including ID, may be more likely to separate/divorce and have lower levels of satisfaction with their partner relationship compared to other parents[17,18]. We developed the 1,000 Families Study to explore these issues further and to extend current knowledge across several domains. This article describes the cohort recruited for the 1,000 Families study. Future publications will concentrate on exploring relevant research questions and report key findings related to family wellbeing.

Study aims

The main aims of the 1,000 Families Study are:

1. To describe the wellbeing of families of children with ID and to compare these data wherever possible with normative data and/or to national datasets (especially the UK Millennium Cohort Study). Family members include mothers (or primary parental caregivers), fathers (or secondary parental caregivers), and siblings.
2. To examine wellbeing differences between mothers and fathers in families of children with ID.
3. To explore correlates of maternal, paternal and sibling wellbeing and relationship quality, including: deprivation and socio-economic circumstances; other child, parent, sibling and family demographic factors; other family members' wellbeing, and the child with ID's behaviour problems and adaptive skills.
4. To explore correlates of the behavioural and emotional wellbeing of the child with ID, including: family deprivation; other child, parent, sibling and family demographic

For peer review only

factors; other family members' wellbeing, the child with ID's adaptive skills, and parenting attitudes and behaviours.

STUDY DESCRIPTION

To date, we have conducted an online cross-sectional survey and telephone interviews of over 1,000 families of children with ID who were living in the UK at the time of recruitment. Recruitment involved a multi-point contact method using websites, social media, advertisements in disability organisations' newsletters, and by approaching special schools and parent support organisations. To examine longitudinal associations, we are currently inviting these same families to participate in further data collection (Wave 2), which includes an online (or postal on request) survey but no telephone interview. To summarise the first and second waves of the 1,000 Families study:

Wave 1 data collection

The primary (and secondary if available) parental caregiver were asked to complete the same online survey: 1,184 primary caregivers and 93 secondary caregivers completed the Wave 1 survey between November 2015 and January 2017. The primary caregiver was also invited take part in a telephone interview following the questionnaire to provide information about the adaptive skills and behaviours of their child with ID. The telephone interview was arranged at a time convenient to the primary caregiver and most often took around an hour. The telephone interviews were completed by 644 primary caregivers. Differences between those who did and did not complete a telephone interview are discussed in Findings to Date.

Wave 2 data collection

Participants for Wave 2 are the caregivers who indicated during Wave 1 that they were willing to be contacted again to be invited to participate in Wave 2. The telephone interview was replaced with an additional measure on adaptive skills in the online survey. Caregivers

For peer review only

are first contacted 2 years and 9 months after they completed Wave 1. Wave 2 data collection began in August 2018, and is anticipated to end in January 2021.

Study funding

The study is funded by Cerebra, a non-profit charity working with families with children with brain conditions. The project has also received funding from the Economic and Social Research Council Warwick Doctoral Training Centre, and the University of Warwick via PhD scholarship funding.

Study population

Inclusion criteria

1. For Wave 1, families with one or more children aged between 4 years and 15 years 11 months old who have an ID as reported by a parental caregiver. The child age range was initially (from November 2015) specified as 4 years to 11 years. In February 2017, an ethics amendment allowed us to expand recruitment to children aged up to 15 years and 11 months to meet families' interest in the study and to reach our sample target of over 1,000 families.
2. Families with at least one parental caregiver responding to the survey.
3. Families who live within the UK (i.e. England, Northern Ireland, Scotland or Wales).

Exclusion criteria

1. For Wave 1, families in which the child with ID lives outside of the family home on a full time basis (e.g., in a 52 week residential school placement).
2. Parental caregivers whose English literacy skills would not allow them to participate in an online survey and telephone interview.
3. Parental caregivers under 18 years old.

Wave 1 Cohort Description

For peer review only

Tables 1 and 2 summarise the key socio-demographic variables of the cohort child and their family respectively at Wave 1. All data were reported by the primary caregiver of the child with ID. These were usually the biological mother of the child with ID (78.0%) or another female relative (84.9% of the primary caregivers were female). Cohort children with ID were aged between four years and 15 years and eleven months, with a mean age of 9.01 (SD = 2.93). There were more boys than girls (61.8% were male; 27.0% were female; 11.1% missing). The vast majority of families lived in England (*n* = 1,031), with fewer living in Scotland (*n* = 48), Wales (*n* = 83), and Northern Ireland (*n* = 9).

Table 1 provides some data indicative of ID severity in the cohort children. In terms of level of ID, 42.3% were reported by parents to have a severe to profound ID, and 42.2% a mild to moderate ID (15.5% missing). A mild to moderate ID was defined as "children with a mild to moderate intellectual disability can typically communicate and look after themselves well, but may take a bit longer to learn new skills compared to other children of the same age". A severe to profound ID was described as "children with a severe to profound intellectual disability are likely to have complex and multiple difficulties which require extensive support to learn and carry out daily activities". Inclusion policies in the UK schooling context means school-type is not precisely indicative of children's needs, particularly in primary schooling, nonetheless, 39.1% of children were attending a special school, in addition to 12.8% attending a mainstream school in a special provision or unit, and 28.1% attending a mainstream school. The Vineland Adaptive Behaviour Composite scores ranged from 25-100, with the mean score being 57.97 (*SD* = 11.13). The range of standard scores on the Vineland domains of Communication (25-117; mean = 62.02; *SD* = 13.63), Daily Living Skills (25-103; mean = 57.53; *SD* = 12.41), and Socialisation (15-110; mean = 59.24; *SD* = 12.40) were similarly wide. Thus, the full range of ID severity is represented within this cohort.

For peer review only - http://bmjopen.bmj.com/site/about/guidelines.xhtml

Common co-occurring conditions in the cohort of children with ID included autism (45.5%) and Down syndrome (13.6%). Other diagnoses were less common, but included Fragile X, Angelman syndrome, and numerous rare genetic conditions. A large number of children had additional physical health conditions or sensory impairments, including: visual impairments (23.9%), hearing impairments (13.9%), epilepsy (12.5%), mobility problems (49.3%) and 'other physical health problems' (41%).

Measures

The study captured information on parental positivity, parental mental health, care burden/stress, life satisfaction, parent relationship, family satisfaction, parenting, child behavioural and emotional wellbeing, child adaptive behaviour, sibling behavioural and emotional wellbeing and sibling relationship quality. Tables 3 and 4 summarise the study measures, with number of missing items for each variable during Wave 1 indicated.

Patient and public involvement

The survey content and method was designed collaboratively with Cerebra, an organisation that supports and advocates for families of children with developmental disabilities, including ID. The final author (JM) participated in the design of the survey in her capacity as Cerebra staff member and PhD student at the University of Warwick. To date, the research team and Cerebra have collaborated to disseminate findings to families of children with ID through parent and practitioner research seminars that Cerebra regularly hosts.

Ethics

The study is being conducted in accordance with the ethical principles of the British Psychological Society. Approval was obtained from the National Health Service (NHS) West Midlands – South Birmingham Research Ethics Committee prior to commencement of the study. Sponsor approval was also obtained from the University of Warwick. There are no significant risks for the families from taking part in this study, but as some of the questions

For peer review only

included in the survey ask about potentially challenging issues, such as parental and child wellbeing and difficulties, signposting to various resources and helplines has been provided in the information sheet and at the end of the survey for caregivers.

All participant information is stored in line with the requirements of the Data Protection Act 1998. While this study was approved and began before changes to the General Data Protection Regulation (GDPR) in 2018, data collection and procedures in Wave 2 are in line with GDPR requirements. Caregivers are informed through information sheets and

For peer review only

**Table 1.** Socio-economic and demographic information for Wave 1 Cerebra 1,000 Families sample: Child with ID	
**Child**	
Child age range (years)	4.01-15.92
Child age: mean (SD) (years)	9.01 (2.93)
Missing age n	175 (14.8%)
Child gender:	
n of males	732 (61.8%)
n of females	320 (27.0%)
Missing gender n	132 (11.1%)
Parental caregiver reported ID Level	
Severe to profound ID	501 (42.3%)
Mild to moderate ID	500 (42.2%)
Missing information	183 (15.5%)
Parental caregiver reported Autism diagnosis of child	539 (45.5%)
Down syndrome	161 (13.6%)
Cerebral palsy	87 (7.3%)
Child has a visual impairment	
Yes	283 (23.9%)
No	719 (60.7%)
Missing information	182 (15.4%)
Child has a hearing impairment	
Yes	164 (13.9%)
No	847 (71.5%)
Missing information	173 (14.6%)
Child has epilepsy	
Yes	148 (12.5%)
No	861 (72.7%)
Missing information	175 (14.8%)
Child has mobility problems	
Yes	584 (49.3%)
No	422 (35.6%)

For peer review only

Missing information	178 (15.0%)
Child has other physical health problems	
Yes	485 (41%)
No	523 (44.2%)
Missing information	176 (14.9%)
Vineland Adaptive Behaviour Composite (ABC)	
Mean (SD)	57.97 (11.14)
Range	25-100
Total responses	640 (54.1%)
Missing information	544 (45.9%)
Vineland Communication standard score	
Mean (SD)	62.02 (13.63)
Range	25-117
Total responses	642 (54.2%)
Missing information	542 (45.8%)
Vineland Daily Living Skills standard score	
Mean (SD)	57.53 (12.41)
Range	25-103
Total responses	643 (54.3%)
Missing information	541 (45.7%)
Vineland Socialisation standard score	
Mean (SD)	59.24 (12.40)
Range	15-110
Total responses	643 (54.3%)
Missing information	541 (45.7%)
Type of School:	
n of children attending special school	463 (39.1%)
n of children attending mainstream school	333 (28.1%)
n of children attending mainstream school in either a special unit or special educational needs provision	151 (12.8%)
Missing information	178 (15.0%)

For peer review only

Table 2. Socio-economic and demographic information for Wave 1 Cerebra 1,000 Families sample: Parent and Family

**Primary Caregiver and Family**	
Female Primary Caregiver	1005 (84.9%)
Primary Caregiver Relationship to Child	
Biological Mother	923 (78%)
Adoptive Mother	53 (4.5%)
Biological Father	46 (3.9%)
Grandmother	13 (1.1%)
Missing information	129 (10.9%)
Primary Caregiver Employment status:	
In a job working for an employer	391 (33.0%)
Looking after home and family	388 (32.8%)
Self-employed	103 (8.7%)
Doing something else	100 (8.4%)
Missing information	129 (10.9%)
Primary caregiver education level:	
Degree level	465 (39.3%)
Higher education but below degree level	253 (21.4%)
A/AS Levels or equivalent	104 (8.8%)
5 or more GCSEs at A-C or equivalent*	74 (6.3%)
Some GCSEs passes or equivalent	93 (7.9%)
No qualifications	13 (1.1%)
Missing information	179 (15.1%)
Primary caregiver relationship status:	
Married and living with spouse/civil partner	631 (53.3%)
Divorced/separated/single/widowed/not currently living with partner	226 (19.1%)
Living with partner	141 (11.9%)
Missing information	186 (15.7%)

For peer review only

Primary Caregiver Ethnicity:	
White British	930 (78.5%)
White other (Irish, Travelling community, other)	55 (5.2%)
Asian/Asian British	26 (2.3%)
Black (African/Caribbean/Black British)	15 (1.3%)
Remaining ethnic groups (Mixed / multiple ethnicity, Arabic, Any other ethnic background etc.)	23 (2.0%)
Missing information	135 (11.4%)
Country:	
England	1031 (87.1%)
Scotland	48 (4.1%)
Wales	83 (7.0%)
Northern Ireland	9 (0.76%)
Missing	13 (1.10%)
Family living in most deprived 10% of neighbourhoods based on indices of multiple deprivation (IMD)	90 (7.6%)
Missing IMD information	34 (2.9%)
Child with ID has a sibling aged between 4 and 15 years 11 months	
Yes	612 (51.7%)
No	360 (30.4%)
Missing information	212 (17.9%)
Nearest-in-age sibling of child with ID has a longstanding illness, disability or infirmity	
Yes	168 (27.5%)
No	437 (71.4%)
Missing information (from those who indicated child has sibling in age range)	7 (1.1%)

For peer review only

Table 3. Parent and family measures used within the Cerebra 1,000 Families Study

**Domain**	**Measure**	**Scoring**	**Measured at**	**Wave 1 Measure completeness**
Parental positivity	Positive gains scale [Jess, Bailey, Pit-ten Cate, et al., Measurement invariance of the Positive Gains Scale in families of children with and without disabilities]<br>7 items<br>Perceived benefits for parent (5 items)<br>What family has gained (2 items)	5-point scale from 0 (*strongly agree*) to 4 (*strongly disagree*). Original scale had a lower score indicating higher positive gains. The scoring was reversed so that a higher score indicates higher positive gains.	W1, W2	Complete *n* = 1004<br>Missing *n* = 180
Parent mental health	Kessler 6 (K6)[19]<br>6 items	5-point Likert scale asks caregivers how often they've experienced six symptoms over the last 30 days from 0 (*none of the time*) to 4 (*all of the time*). Total score is calculated by summing the responses. A score of 13 and above indicates serious mental illness[20].	W1, W2	Complete *n* = 1000<br>Missing *n* = 184
Carer burden/stress	'Impact of care-giving on carer'<br>Survey of Informal Carers in Households 2009/2010[21]<br>7 items	Items ask individuals whether certain aspects of their lives have been affected by caring for another (*Yes* or *No*). Totals are generated for the responses, and higher scores indicate higher carer burden/stress.	W1, W2	Complete *n* = 963<br>Missing *n* = 221
Life satisfaction	Life satisfaction scale[22]<br>1 item	Single item measure that asks caregivers to rate their general life satisfaction from 1 (*completely dissatisfied*) to 10 (*completely satisfied*). Scores 6-10 indicate that respondents are 'satisfied overall'. Scores 4 and below indicate that participants are 'dissatisfied overall'.	W1, W2	Complete *n* = 998<br>Missing *n* = 186
Parent relationship – parent's perception of relationship with their partner (if applicable)	Disagreement over issues related to child[23]<br>1 item	This item asks caregivers to report on how often they disagree over issues relating to their child on a 6-point Likert scale (*Never* to *More than once a day*). There is also a response for 'Can't say'.	W1, W2	Complete *n* = 771<br>No live-in partner *n* = 226<br>Missing *n* = 187
	Happiness of relationship scale[23]<br>1 item	Caregivers are asked to rate how happy they are in their relationship from 1 (*very unhappy*) to 7 (*very happy*). There is also a response for 'Can't say'.	W1, W2	Complete *n* = 771<br>No live-in partner *n* = 226<br>Missing *n* = 187
Family functioning	Family APGAR scale[24]<br>5 items<br><br>Adaptability, Partnership, Growth, Affection and Resolve (APGAR)	Respondents are required to rate the frequency of feeling satisfied with each parameter on a 3-point Likert scale, from 0 (*hardly ever) to* 2 (*almost always*). The scale is scored by summing the 5 items. A higher score indicates a greater degree of satisfaction with family functioning (8-10 = *highly*	W1, W2	Complete *n* = 993<br>Missing *n* = 191

For peer review only - http://bmjopen.bmj.com/site/about/guidelines.xhtml

		functional, 4-7 = *moderately dysfunctional*, 0-3 = *dysfunctional*).		
Sibling behavioural and emotional wellbeing	Strengths and Difficulties Questionnaire (SDQ) (Parent questionnaire)[25]<br>25 items<br><br>Subscales: Emotional problems, Conduct problems, Hyperactivity/inattention, Peer relationship problems, Prosocial behaviour	Caregivers are asked to assess the extent to which each statement applies to the child based on the last 6 months using a 3-point rating scale (0 = *not true* to 2 = *certainly true*). The items are divided into 5 subscales. A total difficulties score can be obtained by summing the first four sub-scale scores: *close to average* (0-13), *slightly raised* (14-16), *high* (17-19), and *very high* (20-40).	W1, W2	Complete *n* = 603<br>No sibling *n* = 360<br>Missing *n* = 221
Sibling relationship	Sibling Relationship Questionnaire (SRQ) (Parent questionnaire) (revised)[26]<br>10 items<br>Adapted version using key items from two subscales of SRQ – SF:<br>Warmth and closeness (6 items)<br>Conflict (4 items)	Caregivers are asked about the sibling relationship on a 5-point Likert scale from 1 (*very much*) to 5 (*extremely much*). These items can then be used to derive measures that encapsulate positive or negative aspects of the sibling relationship.	W1, W2	SRQ warmth:<br>Complete *n* = 596<br>No sibling *n* = 360<br>Missing *n* = 228<br>SRQ conflict:<br>Complete *n* =599<br>No sibling *n* = 360<br>Missing *n* = 225
Parenting attitudes and behaviours	Child-parent relationship scale (CPRS)[27]<br>15 items<br>Conflict (8 items)<br>Closeness (7 items)	The caregiver states their feelings and beliefs about their relationship with their child and about the child's behaviour towards them, by responding to statements on a 5-point Likert scale (1 = *Definitely does not apply* to 5 = *Definitely applies*). CPRS generates a separate score for the individual conflicts and closeness constructs by summing the scores in each subscale	W1, W2	Closeness:<br>Complete *n* = 978<br>Missing *n* = 206<br>Conflict:<br>Complete *n* = 983<br>Missing *n* = 201
	Alabama Parenting questionnaire (Parent form)[28]<br>12 items<br>Positive parenting (6 items) Inconsistent discipline (6 items)	Parents/caregivers are asked about their parenting behaviours on a five-point Likert scale (1 = *Never* to 5 = *Always*). The items in each scale are summed to obtain two scale scores.	W1, W2	Positive parenting:<br>Complete *n* = 972<br>Missing *n* = 212<br>Inconsistent discipline:<br>Complete *n* = 961<br>Missing *n* = 223
	Parent-child activities index<br>5 items	This questionnaire was constructed for the purposes of this study. Some of the questions have been used in national UK surveys such as the Millennium Cohort Study, and where relevant, items were adapted to be more appropriate for families of children with ID. The questions ask about parent-child shared activities. Answers are provided on a 5-point Likert scale (from 1 = *Not at all* to 5 = *Every day*).	W1<br>*Removed from W2 due to parental feedback.*	Complete *n* = 974<br>Missing *n* = 210

Note. W1 and W2 indicates Wave 1 and Wave 2

For peer review only - http://bmjopen.bmj.com/site/about/guidelines.xhtml

Table 4. Child measures used within the Cerebra 1,000 Families Study

Domain	Measure	Scoring	Measured at	Wave 1 Measure completeness
Child with ID behavioural and emotional wellbeing	Strengths and Difficulties Questionnaire (SDQ) (Parent questionnaire)[25]<br>25 items<br>Subscales: Emotional problems, Conduct problems, Hyperactivity/inattention, Peer relationship problems, Prosocial behaviour	Caregivers are asked to assess the extent to which each statement applies to the child based on the last 6 months using a 3-point rating scale (0 = *not true* to 2 = *certainly true*). The items are divided into 5 subscales. A total difficulties score can be obtained by adding the first four sub-scale scores and categorising them into a four band system: *close to average* (0-13), *slightly raised* (14-16), *high* (17-19), and *very high* (20-40).	W1, W2	Complete *n* = 985<br>Missing *n* = 199
Behavioural and emotional problems of the child with ID	Development behaviour checklist (parent version) (DBC-P)[29]<br>96 items<br>Telephone interview<br>Subscales: Disruptive/Anti-social Behaviour, Self-absorbed, Communication Disturbance, Anxiety, Social Relating	Items are scored on a 0, 1 or 2 rating scale (0 = *Not true as far as you know*, 1 = *Somewhat or sometimes true*, 2 = *Very true or often true*). The Total Behaviour Problem Score (TBPS) is an overall measure of emotional and behavioural problems, and can detect clinically significant levels of overall emotional and behavioural disturbance (indicated by a score of 46 or greater).	W1	Complete *n* = 644<br>Did not consent to telephone interview *n* = 198<br>Missing *n* = 342
Adaptive behaviour skills of the child with ID	Vineland adaptive behaviour scales – second edition (parent interview form) (VABS- II)[30]<br>30-40 minute telephone interview<br><br>Communication Skills<br>Daily Living Skills<br>Socialisation Skills	An overall composite score, and domain scores, can be derived with reference to age during typical development during which children can perform the task items. Four standardised scores in total were derived for the present research: Adaptive Behaviour Composite, Communication Skills, Daily Living Skills, and Socialisation Skills. Age Equivalency scores were also calculated.	W1	Complete *n* = 645<br>Did not consent to telephone interview *n* = 198<br>Missing *n* = 341
Adaptive behaviour skills of the child with ID	The GO4KIDDS Brief Adaptive Scale[31]<br>9 items	Items cover communication, self-help skills, social interaction, and support needs. Each item is rated on a 5-point scale where higher scores indicate greater skill level and greater independence. These item scores are summed to get an overall Adaptive Behaviour score. We have amended the original GO4KIDDS to include an additional item asking about alternative methods of communication following feedback from caregivers.	W2<br><br>Replaced the VABS-II interview	Not yet known

Note. W1 and W2 indicates Wave 1 and Wave 2

consent forms on what data are collected, why this is necessary, and how these data are stored. All aspects of the study are conducted on the basis of explicit written consent to take part in each stage of the study. All research data are stored electronically and password protected on firewalled University computers with access limited to the research team. The password and access to the research data, including participant information, is restricted to the Chief Investigator and the research team.

For Wave 2 of the data collection, consent is collected as it was in Wave 1 and participants are asked again if they would be willing to be contacted for any future research. At Wave 2, a £10 gift voucher was introduced as a Thank You to caregivers who complete the survey. To mitigate against potential biases arising from the monetary incentive, it is made clear in the information sheet that caregivers do not have to answer any questions that make them feel uncomfortable and they will still receive the voucher.

Dissemination

Throughout the study, results will be published in peer-reviewed academic journals. Presentations of study findings will be made at research conferences and seminars. The charity Cerebra and other stakeholders will be involved in methods of dissemination for reaching the wider community, particularly families of children with ID. Caregivers who took part in the study, and have consented, receive updates about the study through a newsletter and social media, which the public can also access. This allows findings to be summarised and presented in an accessible format in addition to any academic papers or presentations.

COHORT DESCRIPTPTION

The remainder of this manuscript will provide a description of the cohort participating in the 1,000 Families study. No findings have yet been reported from the data on this cohort. Overall, the parent-reported severity of ID, educational setting, and VABS scores indicate that

For peer review only

this cohort includes children across a wide range of ID severity, although the VABS scores indicate more children in the mild-moderate range, but this would be expected given a lower prevalence for severe to profound ID.

Telephone interviews were completed by 644 of the primary caregivers indicating that families who completed both the survey and the telephone interview may have had access to more resources that enable them to contribute more time to research participation. For instance, deprivation levels may be associated with telephone interview participation. To identify family deprivation, we constructed a composite variable with values ranging from zero to four, incorporating measures of: subjective poverty, hardship, income poverty, and neighbourhood deprivation. Primary caregivers that did not complete telephone interviews had a mean deprivation composite score of 1.71 (SD = 1.10) compared to a mean deprivation composite score of 1.37 (SD = 1.09) for primary caregivers that completed a telephone interview ($t(948) = 4.72$; $p < .001$; $d = .32$; MD = 0.35, 95% CI .20, .49). Primary caregivers who completed the telephone interviews were more likely to have a higher education qualification (75.1%) compared to primary caregivers that did not complete a telephone interview (66.6%; $\chi2(1, N = 1{,}002) = 8.58$; $p = .003$; $\phi = .093$). Primary caregivers who were single parents provided a telephone interview in 53.5% of cases, compared to primary caregivers from two-parent households, who provided a telephone interview in 65.3% of cases ($\chi2(1, N = 998) = 10.30$; $p = .001$; $\phi = -.102$.

In addition to resources, differences between those who participated in telephone interviews and those who did not included differences in child profiles. Children of caregivers who did not take part in the telephone interview had higher total SDQ total behaviour scores with a mean score of 22.00 (SD = 6.52) compared to a mean score of 20.63 (SD = 6.48) for those who did participate ($t(983) = 3.201$; p .001; $d = .21$; MD = 1.38, 95% CI .53, 2.22). Primary caregivers who did not complete the telephone interviews had children with ID that

had fewer coexisting physical health problems (such as epilepsy, mobility problems, hearing or visual problems; mean = 1.49; *SD* = 1.22) compared to the children with ID of primary caregivers that did complete the telephone interviews (mean = 1.76; *SD* = 1.33; $t(985) = -3.157$; *p* .002; *d* = .21; *MD* = -0.27, 95% CI -.43, -.10).

Comparison with indicators from the 2011 census of England and Wales[32] indicate lower participation of families identifying as Black, Asian or Minority Ethnic (5.6% of the 1,000 Families sample compared to 13% of the population of England and Wales), but expected numbers of families identifying as White British (78.5% of the 1,000 Families sample, compared to 80.5% in the 2011 Census) (see Table 2 for full ethnicity information on the 1,000 Families). In terms of educational qualifications, the survey sample were more highly educated (39.1% of the primary caregivers in the 1,000 Families were educated to degree level, in comparison to 27% in the England and Wales 2011 Census)[32]. Whilst 23% of the population in England and Wales had no educational qualifications, only 1.1% of the 1,000 Families sample reported having no educational qualifications. These comparisons to the census data should be viewed with caution as the census data about qualifications include the full age-range of adults living in England and Wales in 2011, whereas our sample of primary caregivers disproportionately includes women of childrearing age across all four countries of the UK. Policy changes leading to the expansion of UK higher education in recent decades means we would expect that the primary caregivers in our sample were more likely to have completed degrees than were previous generations. Furthermore, the 2011 census data included people aged 16-18 and this group were less likely to have any qualifications yet, although they may have been in education. In terms of socio-economic indicators, 7.6% of the families in the 1,000 Families sample were living in the most deprived areas in their respective country based on the indices of multiple deprivation for England, Scotland, Wales and Northern Ireland [33-36]. These indices offer a measure of relative

For peer review only

deprivation at a local scale by combining information on income poverty, education, unemployment, health, crime, barriers and access to services, housing, and physical environment for each small area and then rank-ordering areas within each country.

Future directions

The data will be analysed in cross-sectional and longitudinal studies. Where possible, data will be compared with data from normative or population-representative samples. Correlation and regression approaches will be used to explore factors associated with the wellbeing of mothers, fathers, siblings, and the child with ID. Where appropriate, models will account for the nested nature of data within families. The cohort provides scope to explore a range of research questions, for example, studies exploring: associations between behavioural outcomes for children with ID and their siblings in relation to their sibling relationship quality, anxiety in children with ID with and without autism, and wellbeing in mothers of children with Down syndrome. The cross-sectional studies using data from Wave 1 will also help to develop models and questions that can be explored through longitudinal research following future waves.

STRENGTHS AND LIMITATIONS

This study is, as far as we are aware, the largest cohort of families with a child with ID in the UK and will make an important contribution to the research on the wellbeing of children with ID and their families. Particularly important is the longitudinal design which currently includes a second wave of data collection that is underway, with a third wave under development. This adds to the growing global research following families of children with ID and/or autism over time. For example, the Adolescents and Adults with Autism (AAA) Study, is an ongoing, longitudinal study with 406 participants, based in the USA, with its sample recruited from Massachusetts and Wisconsin [37-38]. A strength of the AAA study is that is has followed up families for over 20 years, beginning in 1998. Our study will differ in

For peer review only

that we have recruited participants from across the UK, rather than specific geographic locations within a country. We have also recruited families of children with ID rather than autism specifically, although we do have a large group of children with autism within our sample (n = 539). Selection of measures allows for some comparison with population-representative samples in the UK (e.g. the Millennium Cohort Study[39]). Results will inform parents, clinicians, and educational professionals on factors related to the wellbeing of children with ID, their parents, and their siblings.

Designing this research study via a questionnaire and telephone interview allowed us to recruit a large sample from across the UK. However, this precluded an independent ascertainment of child ID, and/or associated developmental conditions (e.g., autism). Instead, information on the child is based solely on parental report, although this includes their educational setting, parent-reported severity of ID, any diagnoses, and for those who took part in the telephone interview, a measure of child adaptive behaviour. The implication of this limitation is that the survey cannot verify the extent of clinical diagnoses available for each child with ID. Where research evidence has examined the validity of parental reports, it has suggested that parents' reports correspond well to clinical diagnoses available[40, 41] and that parental reports on children's developmental outcomes are accurate[42].

Although the sample size recruited at Wave 1 is large (>1,000 families), the sampling design (convenience sample recruited through social media and ID-related organisations) may have resulted in sampling bias, where families of children with ID in contact with charitable organisations and/or access to social media may be more likely to be included. However, although not fully representative of the UK, the findings discussed previously indicate that the 1,000 Families sample includes elements of diversity that have the potential to provide insights into families with a child with ID from various backgrounds.

For peer review only

Another limitation of this study is that not all primary caregivers completed a telephone interview as well as the survey – 644 out of 1,184 primary caregivers completed a telephone interview. As previously summarised in the Findings to Date section, there were demographic differences between these groups. For example, those primary caregivers that did not take part in the telephone interview had higher levels of poverty, lower levels of qualifications, were more likely to be a single parent, and reported higher average SDQ total behaviour scores for their children with ID. The children of the primary caregivers that did not complete the telephone interviews had fewer co-existing physical health problem labels compared to the children of primary caregivers that did complete the telephone interviews.

Last, although 772 of the primary caregivers in the sample indicated they were married and/or living with a partner, only 93 secondary caregivers participated in the survey. Overall low rates of participation of fathers or secondary caregivers in family research is a well-described phenomenon that may relate to role allocation between parents regarding child care, working commitments, and primary responsibility for contact with disability and educational services[43-47]. The 1,000 Families study directly recruited and encouraged fathers to take part in the study, but it is likely that a recruitment strategy primarily focused on the family was not specific enough to encourage greater participation from fathers and secondary caregivers. There is a need for further investigation on why other family members, particularly fathers, were not as likely to take part, and whether there are recruitment strategies which are more successful to reach fathers and secondary caregivers, as some research has highlighted the importance of inviting fathers directly and emphasizing the importance of their participation[47, 43].

COLLABORATION

Initial data analyses and publications will be generated by researchers at CEDAR, University of Warwick as part of the 1,000 Families study funded by Cerebra. However, the research

For peer review only - http://bmjopen.bmj.com/site/about/guidelines.xhtml

team welcome researchers interested in future collaboration to contact the corresponding author with their expression of interest. Access and analysis of the data is currently only possible via the University of Warwick, due to the ethical approvals in place.

Acknowledgements We would like to thank all the families that participated in this study. We acknowledge the support from Cerebra in funding, developing and recruiting to this study; and the ESRC Doctoral Training Centre at the University of Warwick for funding support in the form of PhD studentships. We acknowledge the contributions of our study administrators, Alison Baker and Diana Smith. Various researchers assisted in conducting telephone interviews, alongside the authors, to collect data for Wave 1 of this study and we acknowledge their support: Latoya Clarke, Farah Elahi, Gemma Gray, Liz Halstead, Sasha Mandair, Louise Rixon, Suzi Scott and Ruth Thomas.

Author Contributions RH and VT conceptualised and co-designed the study, wrote the study protocol, contributed to the drafting of the manuscript for publication, reviewed and revised the manuscript, and read and approved the final manuscript. NH and CM contributed to the design of the study, contributed to the drafting of the manuscript for publication, reviewed and revised the manuscript, and read and approved the final manuscript. MJ, EL and JM contributed to the design of the study, contributed to the original protocol, and read and approved the final manuscript.

Funding statement The authors acknowledge the funding support of Cerebra, UK, the Economic and Social Research Council Warwick Doctoral Training Centre, and the University of Warwick.

Competing Interests Statement None declared.

Patient consent Parental/guardian consent obtained.

For peer review only

Data Availability Statement Data from this study are not available for sharing due to ethical approval requirements. Researchers interested in collaboration should contact the corresponding author with their expression of interest.

Ethics approval: Full ethical approval was granted for this study from the UK National Health Service (NHS) West Midlands – South Birmingham Research Ethics Committee: REC reference number: 15/WM/0267 (11th September 2015). An amendment was granted to extend the age range (7th March 2017). An amendment was granted for changes made to Wave 2 (8th February 2018) and the ethics committee was informed of further minor changes made to the documents for Wave 2 (27th February 2018). Sponsor approval was also obtained from the University of Warwick.

For peer review only

REFERENCES

1. Salvador-Carulla L, Reed GM, Vaez-Azizi, LM, *et al.* Intellectual developmental disorders: towards a new name, definition and framework for "mental retardation/intellectual disability" in ICD-11. *World Psychiatry* 2011;10(3):175-180. doi:10.1002/j.2051-5545.2011.tb00045.x

2. Platt JM, Keyes KM, McLaughlin KA, Kaufman AS. Intellectual disability and mental disorders in a US population representative sample of adolescents. *Psychol Med.* 2019;49(6):952–961. doi:10.1017/S0033291718001605

3. Anderson LL, Larson SA, MapelLentz S, Hall-Lande J. A Systematic Review of U.S. Studies on the Prevalence of Intellectual or Developmental Disabilities Since 2000. *Intellect Dev Disabil.* 2019;57(5):421-438. doi: 10.1352/1934-9556-57.5.421.

4. Singer GH. Meta-analysis of comparative studies of depression in mothers of children with and without developmental disabilities. *Am J Ment Retard* 2006;111:155-169. doi:10.1352/0895-8017(2006)111[155:MOCSOD]2.0.CO;2

5. Emerson E. Mothers of children and adolescents with intellectual disability: Social and economic situation, mental health status, and the self-assessed social and psychological impact of the child's difficulties. *J Intellect Disabil Res* 2003;47:385-399. doi:10.1046/j.1365-2788.2003.00498.x

6. Emerson E, McCulloch A, Graham H, *et al.* Socioeconomic circumstances and risk of psychiatric disorders among parents of children with early cognitive delay. *Am J Intellect Dev Disabil* 2010;115:30-42. doi:10.1352/1944-7558-115.1.30

7. Hayden NK, Hastings RP, Totsika V, *et al.* A Population-Based Study of the Behavioral and Emotional Adjustment of Older Siblings of Children with and without Intellectual Disability. *J Abnorm Child Psychol* Published Online First: 4 February 2019. doi:10.1007/s10802-018-00510-5

For peer review only

8. Cridland EK, Jones SC, Magee CA, *et al.* Family-focused autism spectrum disorder research: a review of the utility of family systems approaches. *Autism* 2014;18:213-222. doi:10.1177/1362361312472261

9. Kovshoff H, Cebula K, Tsai H-WJ, *et al.* Siblings of children with autism: The Siblings Embedded Systems Framework. *Curr Dev Disord Rep* 2017;4(2):37-45. doi:10.1007/s40474-017-0110-5

10. Seligman M, Darling RB. *Ordinary families, special children.* 3rd ed. New York: Guilford Press, 2007.

11. Trivette CM, Dunst CJ, Hamby DW. Influences of family-systems intervention practices on parent-child interactions and child development. *Topics Early Child Spec Educ* 2010;30:3-19. doi:10.1177/0271121410364250

12. Emerson E, Hatton C. Mental health of children and adolescents with intellectual disabilities in Britain. *Br J Psychiatry* 2007;191:493–499. doi:10.1192/bjp.bp.107.038729

13. Totsika V, Hastings RP, Vagenas D. Informal caregivers of people with an intellectual disability in England: Health, quality of life and impact of caring. *Health Soc Care Community* 2017:25:951-961. doi:10.1111/hsc.12393

14. Griffith GM, Hastings RP. "He's hard work, but he's worth it." The experience of caregivers of individuals with intellectual disabilities and challenging behaviour: A meta-synthesis of qualitative research. *J Appl Res Intellect Disabil* 2014;27:401-419. doi:10.1111/jar.12073

15. Toms G, Totsika V, Hastings RP, *et al.* Access to services by children with intellectual disability and mental health problems: Population-based evidence from the UK. *J Intellect Dev Disabil* 2015;40:239-247. doi:10.3109/13668250.2015.1045460

16. Totsika V, Hastings RP, Vagenas D, *et al.* Parenting and the behavior problems of young children with an intellectual disability: Concurrent and longitudinal relationships in a

For peer review only

population-based study. *Am J Intellect Dev Disabil* 2014;119:422-435. doi:10.1352/1944-7558-119.5.422

17. Risdal D, Singer GHS. Marital Adjustment in Parents of Children with Disabilities: A Historical Review and Meta-Analysis. *Res Pract Persons Severe Disabl* 2004;29:95-103. doi:10.2511/rpsd.29.2.95

18. Saini M, Stoddart KP, Gibson M, *et al.* Couple relationships among parents of children and adolescents with Autism Spectrum Disorder: Findings from a scoping review of the literature. *Res Autism Spectr Disord* 2015;17:142-157. doi:10.1016/j.rasd.2015.06.014

19. Kessler RC, Andrews G, Colpe LJ, *et al.* Short screening scales to monitor population prevalences and trends in non-specific psychological distress. *Psychol Med* 2002;32:959-976. doi:10.1017/S0033291702006074

20. Kessler RC, Barker PR, Colpe LJ, *et al.* Screening for serious mental health difficulties in the general population. *Arch Gen Psychiatry* 2003;60:184-189 doi:10.1001/archpsyc.60.2.184

21. The NHS Information Centre, Social Care team. 451845 Survey of Informal Carers in Households 2009/10 Mainstage questionnaire [Internet]. NHS Information Centre; Leeds, 2010. p. 24. Available from: https://files.digital.nhs.uk/publicationimport/pub02xxx/pub02200/surv-care-house-care-ques-eng-2009-2010-apx2.pdf

22. Department for Environment, Food and Rural Affairs (DEFRA). Life satisfaction and other measures of wellbeing in England, 2007-2011 [Internet]. DEFRA; London, 2011. Available from: https://webarchive.nationalarchives.gov.uk/20130123225714/http://www.defra.gov.uk/statistics/files/Statistical-Release-13-April-2011-wellbeing.pdf

For peer review only

23. University of London, Institute of Education, Centre for Longitudinal Studies. Millennium Cohort Study Second Survey, 2003-2005: CAPI Questionnaire Documentation. [Internet]. Centre for Longitudinal Studies; London, 2007. p. 96-97. Available from: http://doc.ukdataservice.ac.uk/doc/5350/mrdoc/pdf/mcs2_capi_questionnaire_documentation_november_2007.pdf

24. Smilkstein G. The Family APGAR: A proposal for a family function test and its use by physicians. *The Journal of Family Practice* 1978;6:1231-1239.

25. Goodman R. The Strengths and Difficulties Questionnaire: A research note. *J Child Psychol Psychiatry* 1997;38:581-586. doi:10.1111/j.1469-7610.1997.tb01545.x

26. Furman W, Buhrmester D. Children's perceptions of the qualities of sibling relationships. *Child Development* 1998;56:448-461. doi:10.2307/1129733

27. Pianta RC. *Child-Parent Relationship Scale*. Charlottesville, VA: University of Virginia, 1995.

28. Frick PJ. The Alabama Parenting Questionnaire. Unpublished rating scale, University of Alabama, 1991.

29. Einfeld SL, Tonge BJ. *Manual for the Developmental Behaviour Checklist: Primary Carer Version (DBC-P) & Teacher Version (DBC-T) (2nd ed.)*. Clayton, Melbourne: Monash University Centre for Developmental Psychiatry and Psychology, 2002.

30. Sparrow SS, Cicchetti DV, Saulnier CA. *Vineland Adaptive Behavior Scales, Survey Forms Manual*. 3rd ed. Circle Pines, MN: AGS Publishing, 2016.

31. Perry A, Taheri A, Ting V, *et al.* The GO4KIDDS Brief Adaptive Scale. *J Appl Res Intellect Disabil* 2015;28:594-597. doi:10.1111/jar.12143

32. Ethnicity in the UK. *GOV.UK website, Ethnicity facts and figures* 2019. https://www.ethnicity -facts-figures.service.gov.uk/ethnicity-in-the-uk. Accessed 9th March 2019.

For peer review only

33. Gill B. *The English Indices of Deprivation 2015: Statistical Release*. London: Department for Communities and Local Government, 2015. https://www.gov.uk/government /statistics/english-indices-of-deprivation-2015. Accessed 16th June 2019.

34. *Introducing the Scottish Index of Multiple Deprivation 2016.* Edinburgh: APS Group Scotland for The Scottish Government, 2016. https://www2.gov.scot.Resource/ 0050/00504809.pdf Accessed 16th June 2019.

35. *Welsh Index of Multiple Deprivation 2014: Executive summary revised.* Knowledge and Analytical Services, Welsh Government, 2015. https://gweddill.gov.wales/docs/statistics /2015/150812-wimd-2014-summary-revised-en.pdf. Accessed 16th June 2019.

36. *Northern Ireland Multiple Deprivation Measures 2017: Technical Report.* Northern Ireland Statistics and Research Agency, 2017. https://www.nisra.gov.uk/sites/ nisra.gov.uk/files/publications/NIMDM%202017_Technical%20Report.pdf. Accessed 16th June 2019.

37. Woodman AC, Smith LE, Greenberg JS, *et al.* Change in autism symptoms and maladaptive behaviors in adolescence and adulthood: the role of positive family processes. *J Autism Dev Disord* 2015;45(1):111-26. doi: 10.1007/s10803-014-2199-2.

38. Smith LE, Greenberg JS, Mailick MR. The family context of autism spectrum disorders: influence on the behavioral phenotype and quality of life. *Child Adolesc Psychiatr Clin N Am*. 2014;23(1):143–155. doi:10.1016/j.chc.2013.08.006

39. University of London, Institute of Education, Centre for Longitudinal Studies.. Millennium Cohort Study: Fourth Survey, 2008. [data collection]. 7th Edition. UK Data Service, 2017. SN: 6411. doi:10.5255/UKDA-SN-6411-7

40. Daniels AM, Rosenberg RE, Anderson C, *et al.* Verification of parent-report of child autism spectrum disorder diagnosis to a web-based autism registry. *J Autism Dev Disord* 2012;42(2):257-265. doi:10.1007/s10803-011-1236-7

For peer review only

41. Lee H, Marvin AR, Watson T, *et al*. Accuracy of Phenotyping of Autistic Children Based on Internet Implemented Parent Report. *Am J Med Genet **B** Neuropsychiatr Genet* 2010;0(6):1119–1126. doi:10.1002/ajmg.b.31103

42. Charman T, Howlin P, Berry B, *et al*. Measuring developmental progress of children with autism spectrum disorder on school entry using parent report. *Autism* 2004;8(1):89-100. doi:10.1177/1362361304040641

43. Costigan, CL, Cox, MJ. Fathers' participation in family research: is there a self-selection bias? *J Fam Psychol* 2001;15(4):706-720.

44. Cassano M, Adrian M, Veits G, *et al.* The inclusion of fathers in the empirical investigation of child psychopathology: an update. *J Clin Child Adolesc Psychol* 2006;35(4):583-589. doi:10.1207/s15374424jccp3504_10

45. Mitchell SJ, See HM, Tarkow AKH, *et al.* Conducting studies with fathers: Challenges and opportunities. *Appl Dev Sci* 2007;11(4):239–244. doi:10.1080/10888690701762159

46. Doyle O, Weller BE, Daniel SS, *et al.* Overcoming Barriers to Fathers' Participation in Clinically Relevant Research: Recommendations from the Field, *Social Work Research* 2016;40(4):260–264. doi:10.1093/swr/svw015

47. Phares V, Lopez E, Fields S, *et al*. Are fathers involved in pediatric psychology research and treatment? *J Pediatr Psychol* 2005;30(8):631-643. DOI: 10.1093/jpepsy/jsi050

For peer review only
